# In-Depth Aroma and Sensory Profiling of Unfamiliar Table-Grape Cultivars

**DOI:** 10.3390/molecules23071703

**Published:** 2018-07-12

**Authors:** Yusen Wu, Wenwen Zhang, Shuyan Duan, Shiren Song, Wenping Xu, Caixi Zhang, Bhaskar Bondada, Chao Ma, Shiping Wang

**Affiliations:** 1 Department of Plant Science, School of Agriculture and Biology, Shanghai Jiao Tong University, 800 Dongchuan Road, Minhang District, Shanghai 200240, China; senwy886@sjtu.edu.cn (Y.W.); zwwjy0222@163.com (W.Z.); hongloudsy@sjtu.edu.cn (S.D.); sr.song@sjtu.edu.cn (S.S.); wp-xu@sjtu.edu.cn (W.X.); acaizh@sjtu.edu.cn (C.Z.); 2 Wine Science Center, Washington State University, Richland, WA 99354, USA; 3 Institute of Agro-food Science and Technology/Key Laboratory of Agro-products, Processing Technology of Shandong, Shandong Academy of Agricultural Sciences, Jinan 250100, China

**Keywords:** table grape, unfamiliar cultivars, volatile compounds, aroma liking, aroma combinations, aromatic series

## Abstract

We present an in-depth analysis of aroma profiles and sensory attributes, employing solid-phase microextraction gas chromatography/mass spectrometry (SPME-GC-MS) to identify the key compounds driving consumer preference in 19 unfamiliar cultivars. In combination with popular cultivars, we identified a total of 100 compounds in all table grapes, of which 26 key volatiles were correlated with consumer liking. Based on this relationship, five aroma combinations (AC) were formulated, wherein 33 compounds contributed to aroma intensity, and thus, were viewed as active volatiles. The fruity, floral, and sweet aromas were further divided into secondary aromatic series, of which the apple, citrus, orange, rose, geranium, violet, and honey aromas constituted the predominant series in unfamiliar cultivars. Xiangyue and Heikuixiang emerged as the preferred table grapes according to our analysis. By comparison, the popular cultivars showed relatively fewer volatiles, but their contents were much greater than the large number of volatiles identified in the unfamiliar cultivars.

## 1. Introduction

The flavor of any food is the sum of interactions between taste and odor (smell), thus determining its acceptability and preference [1,2,3,4]. This is particularly true of fruits and vegetables, whose consumption is based on consumers’ functional cognition and sensory perception [5,6]. For fruits such as table grapes, sugars and acids activate taste receptors, while a diverse set of volatiles activate olfactory receptors [7,8]. Unlike the taste components, the volatiles are secondary metabolites such as esters, terpenes, etc. These volatiles, although of nutritive value, mainly influence consumer liking [5]. Many volatiles are present in fruits and vegetables; however, not all of them influence consumer liking (retronasal olfaction). For instance, only 29 volatiles of tomato correlated with consumer liking [8]. The same is true of grapes (*Vitis vinifera* L.), a high-value fruit consumed worldwide as fresh fruit (table grapes), and in processed products such as juice and wine [9,10]. Since a rich aroma is essential for grape quality [11,12,13], numerous studies characterized its flavor. However, most of these studies focused on wine grapes and the resulting wines ensuing from various cultivars [14], training systems [15], developmental stages [16], aromatic maturities [17], positions of berries [11,18], microclimates [19], cultural practices such as leaf removal [20,21] and cluster thinning [22], and types of grapes grown for making ice wine [23]. 

Worldwide production volumes of table grapes are enormous, as evident in grape production from China. Although wine consumption in China increased over the last decade, the grape industry is still predominantly based on table grapes, accounting for 80% of total grape production [10]. There exists a plethora of table-grape cultivars; however, the industry is governed by only a few consumer-preferred cultivars such as Kyoho and Jumeigui, whose plantings are increasing. The same is probably true for other countries leading in table-grape production. Such obsessive planting leaves no room for promoting unfamiliar cultivars, even though they might appeal to consumer likings. Despite increased plantings and consumer preference, the consumption of these well-liked table grapes is static [10]. One way to go about this issue is to look for novel aromas to improve flavor by breaking the monotonous plantings (e.g., Kyoho and Jumeigui), and by accentuating plantings of a new core of unconventional cultivars. Thereafter, the chemicals that make the most important contributions to flavor and consumer liking can be identified. Regrettably, except for our previous study that characterized aroma profiles of popular cultivars and their relationship to consumer preference [10], aroma studies in general are rare for table grapes, let alone the unfamiliar cultivars. Other studies that dealt with aroma in table grapes included technological parameters [24], optimizing the harvest dates [25,26], predicting the Muscat aroma [27], and the evolution of volatiles during ripening [28,29], rather than aroma characteristics preferred by consumers. Even though our previous study performed a comprehensive analysis of flavor chemistry, it had one major drawback, and that was describing the aroma profile using only the primary aromatic series. Consequently, a well-characterized relationship between flavor profile and consumer liking could not be ascertained. To address this deficiency, we devised a secondary aromatic series, and applied it to the unfamiliar cultivars, not only to fine-tune the flavor–consumer relationship, but also to overall improve the consumption of table grapes in China, as well as worldwide. Instead of using a consumer panel, we used our popular table-grape data [10] to predict consumer-liking scores for the unfamiliar cultivars. This was accomplished via regression modeling methods, which are routinely adopted to predict liking scores in food and metabolism studies [30,31]. 

Nineteen unfamiliar table grapes were selected for an in-depth analysis of free aroma compounds in both skin and pulp juice, using solid-phase microextraction (SPME) coupled with gas chromatography/mass spectrometry (GC-MS) analyses. We built regression models to predict the relationship between consumer-liking scores and the volatile contents of table grapes. Such analyses were used to (1) describe and distinguish specific aroma profiles using primary and secondary aromatic series (e.g., floral, fruity, and sweet); (2) identify consumer-preferred cultivars by predicting their liking scores; (3) ascertain key aromatic compounds related to the predicted aroma consumer liking; and (4) test the validity of the regression model by comparing volatile contents and liking scores with that of aromatic series values and liking scores. Finally, the above four analyses were used to characterize the overall aroma of table grapes.

## 2. Results

### 2.1. Grape Maturity and Harvest

**Figure 1:**
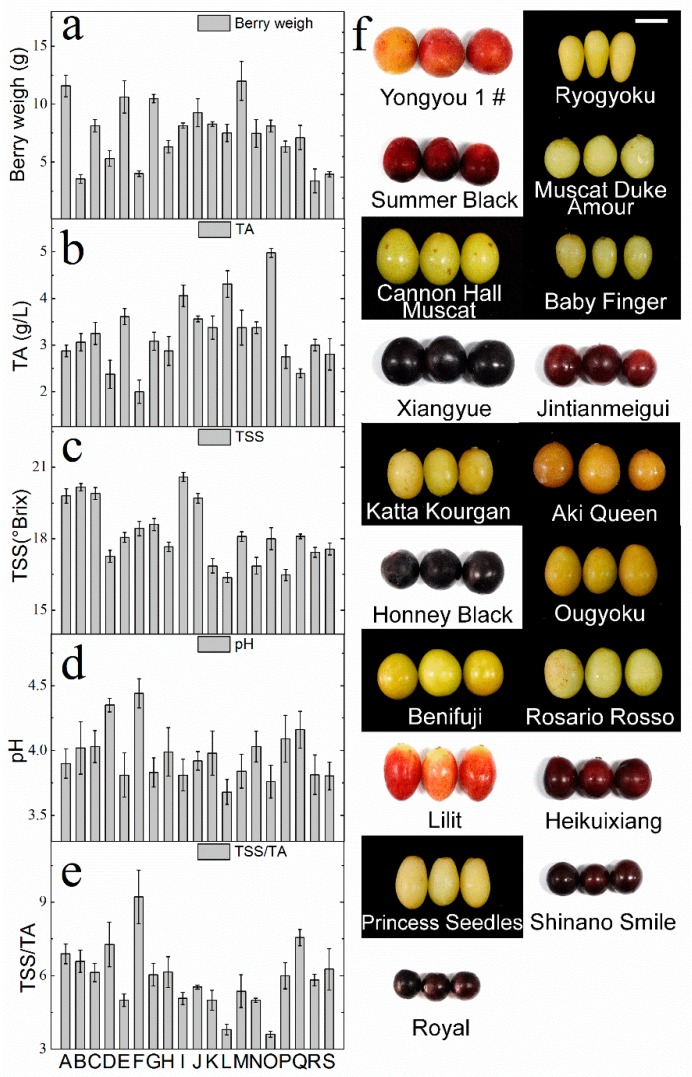
The standard chemical indices of unfamiliar table-grape cultivars. (**a**) Berry weight, (**b**) titratable acidity (TA; g/L), (**c**) total soluble solids (TSS; °Brix), (**d**) pH, and (**e**) sugar:acid ratio (TSS/TA). The photographs (**f**) in the right panel show berries of various grape cultivars. The unfamiliar cultivars include A—Yongyou 1 #, B—Ryogyoku, C—Summer Black, D—Muscat Duke Amour, E—Cannon Hall Muscat, F—Baby Finger, G—Xiangyue, H—Jintianmeigui, I—Katta Kourgan, J—Aki Queen, K—Honey Black, L—Ougyoku, M—Benifuji, N—Rosario Rosso, O—Lilit, P—Heikuixiang, Q—Princess Seedless, R—Shinano Smile, and S—Royal. Scale bars: 25 mm.

The standard chemical attributes are shown in Figure 1. According to the International Organisation of Vine and Wine (OIV) resolution VITI 1/2008 [32], table grapes are considered ripe with total soluble solids (TSS) ≥16 °Brix. In all cultivars, the titratable acidity (TA) ranged from 2–4.98 g/L. The TSS/TA ranged from 5–8, except for those of O (3.62), L (3.80), and F (9.22). Most importantly, the TSS values were higher than 16 °Brix, which indicated that all grape berries reached their optimal maturity [33], and the berries were ready for aroma analysis.

### 2.2. The Volatile Compounds of Unfamiliar Cultivars

**Figure 2:**
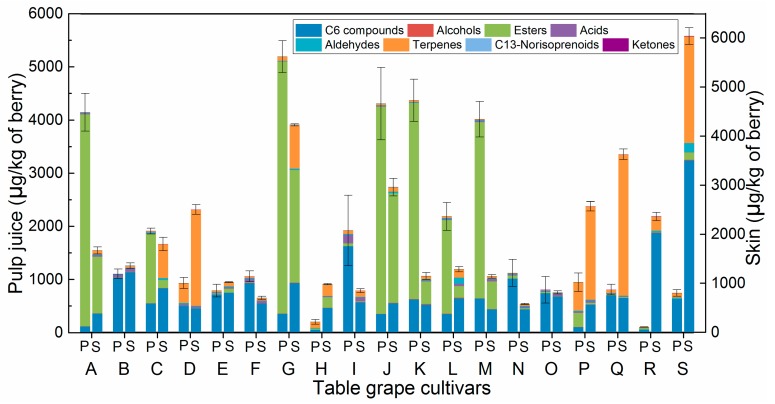
Each class of volatile compound as measured in the pulp juice (P) and skin (S) of unfamiliar table-grape cultivars. The results are shown as mean values. The error bars represent the standard deviation (*n* = 3 biological replications). Capital letters refer to table-grape cultivars, as listed in Figure 1.

Ninety-nine volatiles (72 in both pulp and skin, 11 only in pulp, and 16 only in skin) were identified in whole berries (Tables S1 and S2). The number of alcohols (11) and esters (20) in pulp were greater than that in skin (8 and 19, respectively), whereas an opposite trend occurred for the number of aldehydes (9) and terpenes (27), whereby their numbers were low in pulp juice compared to those in skin (14 and 30, respectively). Five acids, three C_13_-norisoprenoids, and seven C_6_ compounds were found in both skin and pulp. Moreover, two ketones were also found in unfamiliar cultivars. The number of volatiles significantly differed between skin and pulp juice (Figure 2). Esters existed primarily in pulp, whereas the other volatiles mostly occurred in the skin, especially for the terpenes. For instance, in pulp juice (Table S1; Figure 2), esters were the predominant volatiles (69–96%) with ethyl acetate occurring as the primary compound (68–96% of total esters) in seven cultivars (A, C, G, J, K, L, and M; see Figure 1 for cultivars corresponding to upper-case letters). Particularly, P showed relatively high levels of ethyl acetate, which is typical for any cultivar derived from the Kyoho grapevine [10]. On the other hand, terpenes were the predominant volatiles in the skin of three cultivars (D, P, and Q), which accounted for more than 70% of the total content (Table S2). Furthermore, C_6_ compounds were the major volatiles (more than 49%) in the pulp juice of ten cultivars (e.g., B) and in the skin of twelve cultivars (e.g., C). Other compounds were present, but their contents were lower (<200 μg/kg).

The compounds that were detected in popular cultivars (68) were also present in unfamiliar cultivars. However, an additional 22 and 28 volatiles from pulp and skin, respectively, not detected in popular cultivars [10] were detected in the unfamiliar cultivars. So, in total, 99 compounds were detected in unfamiliar cultivars (Tables S1 and S2). On the other hand, four volatiles (2-heptanol, ethyl isobutyrate, ethyl 3-methylbutanoate, and rose oxide II (cis)) identified in the popular cultivars’ skin were present only in the pulp (three of the four volatiles) of unfamiliar cultivars. Because of their low thresholds (Tables S3 and S4), their impact on berry aroma is expected to be negligible. Among all compounds identified, C_6_ compounds constituted the basic background volatiles in both groups (popular and unfamiliar table grapes). The esters primarily existed in pulp juice, whereas all other volatiles mostly occurred in the skin. Nevertheless, qualitative and quantitative differences occurred between these two groups. The number of volatiles found in unfamiliar cultivars was higher than that in the popular cultivars, but the amounts were always high in the popular cultivars (Table S5). This feature, coupled with other traits such as high yield, appearance, and fruit quality attributes, rendered them popular. On the other hand, from an aroma quality perspective, breeders prefer cultivars with high-aroma content, as well as intensity. However, some volatiles may not be present because of the loss of their alleles during the breeding process, which leads to emphasizing the other traits of yield and appearance [8]. Perhaps this is why the popular cultivars showed a low number of aromas but greater contents than the unfamiliar cultivars. Except for terpenes, there was some variation in the content of unfamiliar cultivars; however, the percent change was very small (about 10% or less compared to the popular cultivars). For example, pulp juice contained high contents of various C_6_ compounds with minimum variation (6.25%). The other scenario involved low contents with relatively high variation. This was true of various alcohols in the skin. The total amount was only 2.79 μg/kg, but the percent variation was high (70%). These patterns could be due to genetic differences between the two varieties, which warrants further research. Also, the skin terpenes showed a large variation, especially in popular cultivars. Their content increased by 239.19% (1482.72 μg/kg), whereas geranic acid increased by 255.26% (1028.70 μg/kg; Table S6). Such increases impacted terpene amounts, especially at the whole-berry level (Table S5). Between the two groups, the unfamiliar cultivars are expected to be less floral due to their low terpene amounts (Table S7).

### 2.3. Odor-Active Compounds of Unfamiliar Table Grapes

To identify the true aroma contributors, we determined the odor activity values (OAVs). Thirty-one volatiles (22 in pulp and 27 in skin) existed as aroma-active compounds (OAVs > 1; Tables S3 and S4). Among these, C_6_ compounds, terpenes, esters, and C_13_-norisoprenoids were the dominant contributors, including hexenal and (*E*)-2-hexenal, ethyl hexanoate, d-limonene, rose oxide I (trans), linalool, cedrol, geranic acid, β-damascenone, and β-ionone. For instance, hexenal and (*E*)-2-hexenal were the most active C_6_ compounds contributing to flavors in all samples, except for the pulp of H and R cultivars. Furthermore, the pulp of J, K, and L cultivars showed high values for ethyl butyrate and ethyl 2-methylbutanoate. The OAVs of these compounds were greater than 100. Conversely, ketones and acids were low in content, and hence, showed no odor (<1).

### 2.4. Primary and Secondary Aromatic Series and Aroma Fingerprints

**Figure 3:**
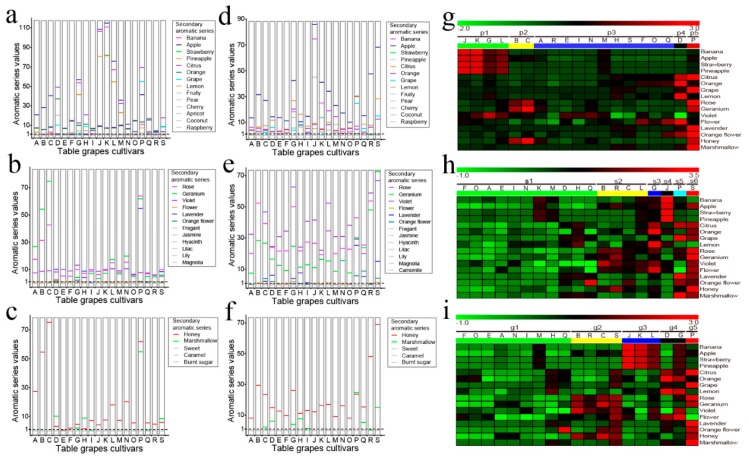
The secondary aromatic series values for pulp juice, (**a**) fruity, (**b**) floral, and (**c**) sweet; and for skin, (**d**) fruity, (**e**) floral, and (**f**) sweet. The values are odor activity values (OAVs) of the aromatic series. The values for rose, banana, apple, strawberry, and pineapple of pulp juice, and rose of the skin represent half of the original values. The gray secondary aromatic series represent values less than 1, and hence, no active aroma. (**g**–**i**) The heatmaps indicate the active secondary aromatic series of every cluster obtained from hierarchical cluster analysis (HCA) analysis (Figure S2, (**a**) pulp juice, (**b**) skin, and (**c**) whole berries). Bars represent mean (*n* = 3 biological replications). The bottom upper-case letters represent the unfamiliar table-grape cultivars, as listed in Figure 1.

The unfamiliar table-grape berries were best characterized by herbaceous, fruity, sweet, floral, and balsamic series (Figures S1a,b). Of these, three series (floral, fruity, and sweet) were rather broad and diverse, and hence, describing their sensorial character was challenging. To tackle this issue, we devised a secondary aromatic series (Figure 3). As per the secondary series, pulp juice distinguished itself by bearing 14 fruity, 12 floral, and five sweet aromas, whereas the skin entailed 13, 13, and five descriptors, in the same order (Figure 3). The descriptors influencing sensorial properties included eight aromas for fruity (e.g., banana), six for floral (e.g., rose), and two for sweet (honey and marshmallow). Among these series, apple, citrus, orange, rose, geranium, violet, and honey were the predominant series (Figure 3). For instance, fruity aromas (Figure 3a) entailing apple, citrus, and orange were dominant in the pulp of most cultivars. On the contrary, the skin was not so fruity. In addition, the P cultivar’s pulp showed high values (54.72) for lavender (Figure 3b), whereas marshmallow dominated only in five pulp (e.g., D) and eight skin (e.g., P) samples (Figure 3c,f).

To reveal the relationship between the cultivars and 16 active secondary series, we performed hierarchical cluster analysis (HCA) and heatmap analysis (Figure 3g–i and Figure S2). The aroma OAV profiles of pulp juice, skin, and whole berries were divided into five, six, and five clusters, respectively (Figure S2). Groups p3, s1, and g1 showed a low level of most secondary flavors, indicating a mild aroma. Groups p1, s4, and g3 had the highest values for four fruity series (banana, apple, strawberry, and pineapple). Groups p4 (D), s3 (G), and g4 (D and G) showed strong aromas of the citrus, orange, lemon, and flower series. Groups p2, s2, and g2 exhibited the highest values in the rose, geranium, and honey series, while groups p5 (P), s5 (P), s6 (S), and g5 (P) were enriched with aromas from the citrus, grape, rose, geranium, lavender, orange flower, honey, and marshmallow series (Figure 3g–i).

**Figure 4:**
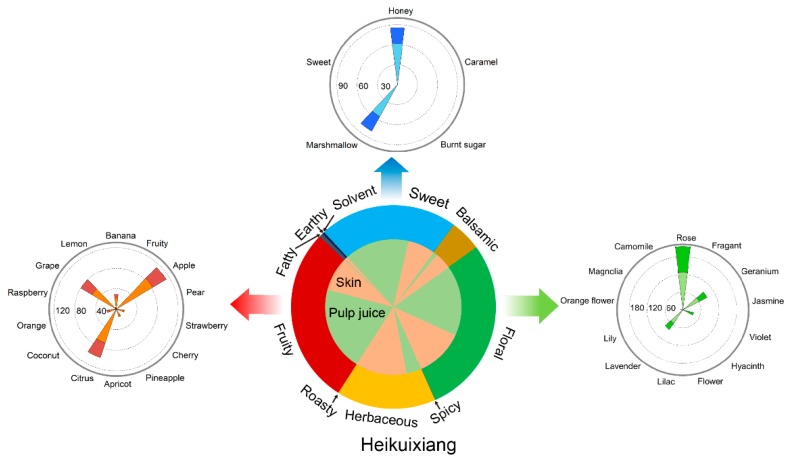
Aromatic fingerprints of Heikuixiang. In the central pie chart, the outer band represents the primary aromatic series, while the interior band represents the berry components—skin (orange) and pulp juice (green). The concentric circles to the left, right, and top of the pie chart represent the fruity, floral, and sweet secondary aromatic series, respectively. In these circles, the outer radial sectors (deep colors) represent the skin, and the inner sectors (light colors) represent pulp juice.

To make the aroma OAV profiles more efficient, we added secondary series to the aroma fingerprints (OAV aroma wheel). With this addition, the consumers could easily make sharp distinctions among different cultivars. For example (Figure 4), P mainly showed four primary aromatic series: sweet, floral, fruity, and herbaceous. The first three aromas were found in pulp, whereas the last one was mostly present in the skin. With regard to the secondary series, it mainly consisted of honey, marshmallow, rose, apple, citrus, and grape aromas. 

### 2.5. Regression Model to Predict Liking Scores

Obtaining consumer-liking scores from the market and sensory evaluations was not possible with the unfamiliar grapes due to their low yields, small plantings, and the fact that they were new to the market. To tackle this problem, we developed an orthogonal partial least squares (OPLS) model using the the popular cultivars [10] to relate the volatile contents (X) with the liking scores (Y). This model was used to predict the consumer-liking scores of unfamiliar cultivars. Although all three models functioned well (*R*^2^Y, *Q*^2^Y > 0.5), the values of *R*^2^Y and *Q*^2^Y were larger than those of the other two models. For model entailing the whole-grape berry, the *R*^2^ values (coefficients of correlation) were greater than those in the other two models. This indicated that the whole-berry model was better than that of either the skin or the pulp juice, and that the unfamiliar cultivars’ liking scores could be predicted well using the popular cultivars’ data.

**Figure 5:**
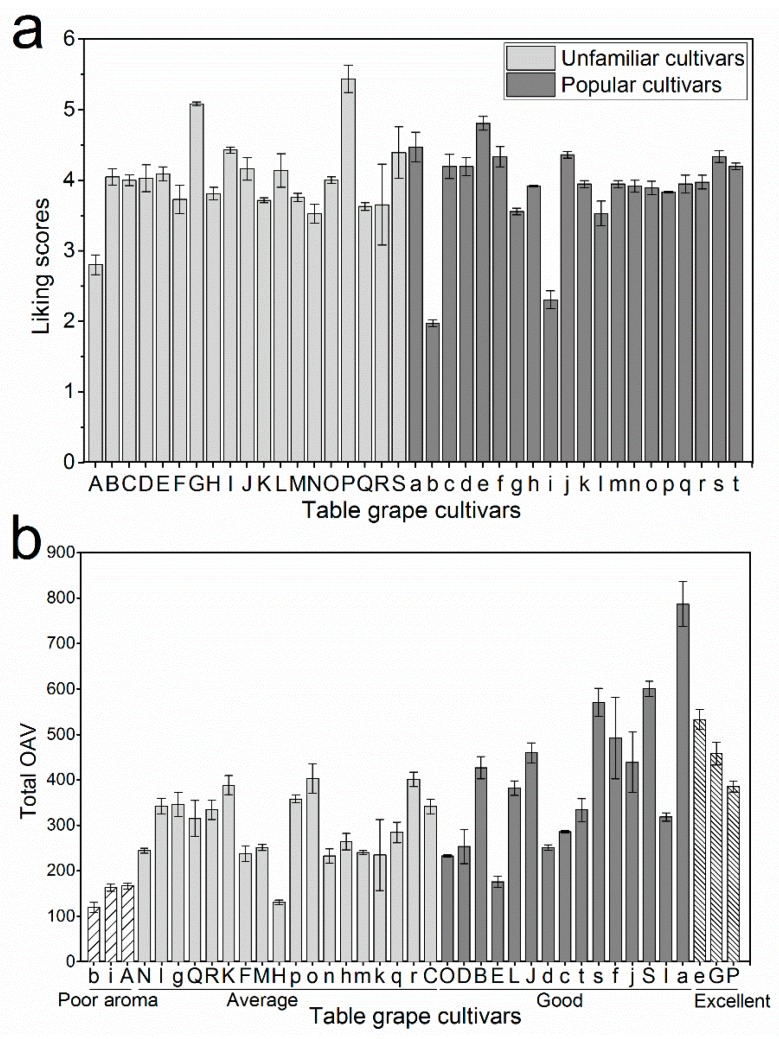
The predicted aroma consumer liking and aroma intensity (OAVs) of all table grapes. (**a**) The predicted aroma consumer-liking scores of unfamiliar and popular cultivars. The scores of unfamiliar cultivars were obtained by orthogonal partial least square (OPLS) regression prediction, whereas the scores for popular cultivars were obtained from sensory analysis in our previous study [10]. Lower-case letters represent popular cultivars: a—Jumeigui, b—Fujiminori, c—Italian, d—Seto Giants, e—Shine Muscat, f—Red Alexandria, g—Rizamat, h—Zuijinxiang, i—Heibaladuo, j—Yoho, k—Oriental Star, l—Kyoho, m—Suiho, n—Black Swan, o—Jingya, p—Black Beet, q—Gold Finger, r—High Bailey, s—Tamina, and t—Centennial Seedless. (**b**) The odor activity values (OAVs) of four categories of table-grape berry (poor, average, good, and excellent aroma). Data represent means ± SD (*n* = 3 biological replications). Upper-case letters indicate table-grape cultivars, as listed in Figure 1.

By excluding strong outliers with the aid of principal component analysis (PCA; Figure S3), an OPLS model was established for the volatiles of pulp juice, the skin, and the whole berry. Among these three (Table 1) models, the whole berry showed a much better fit and predictability (*R*^2^Y = 0.942, *Q*^2^Y = 0.834) than the skin and the pulp juice. The model predicted liking scores with high precision, as evidenced by a root-mean-square error of cross-validation (RMSECV) score of 0.268, which represented about 5% of the full sensory score. The *R*^2^ and *Q*^2^ values of the original model were always higher than the corresponding “permuted” values. Furthermore, the permutation test showed the Q^2^ intercept to be negative (Figure S3i). These data demonstrated that the model aptly fitted to whole berries, and it so happens that people generally consume whole berries, rendering the model as the ideal choice for predicting liking scores. To investigate the differences in liking scores among the cultivars, we performed ANOVA (Table S8), and found that the unfamiliar cultivar A received the lowest score, whereas Xiangyue (G) and Heikuixiang (P) obtained the highest scores (Figure 5a). The scores of other unfamiliar cultivars ranged from 3.5 to 4.5. 

### 2.6. Key Aroma Compounds Associated with Consumer-Liking Scores

To identify the key volatiles associated with liking scores, we further analyzed for the variable importance in projection (VIP) index. Of the 99 compounds identified in unfamiliar table grapes, only 26 functioned as the key compounds contributing to predicted aroma consumer likings (Table S9). These included five aldehydes, 17 terpenes, one acid, one C_13_-norisoprenoid, and two C_6_ compounds, of which β-ionone was the most important compound (VIP = 1.98). The regression coefficient showed negative relationships with liking scores for nine compounds—two aldehydes and seven terpenes. Terpinolene was the most important volatile (VIP = 1.63), followed by octanal (VIP = 1.60). The remaining 17 compounds positively related to the liking scores. β-Ionone showed the highest value for VIP (1.98), and hence, emerged as the most important compound.

### 2.7. Relationship between Aromatic Series and Consumer Liking

In addition to the relationship between liking scores with volatile contents, these scores could also relate to the aromatic series. Such a relationship could be ascertained by developing a model. After performing PCA and running a diagnostic test (Figure S4), we found that the model was ineffective in relating aromatic series with liking scores (*R*^2^Y = 0.499, *Q*^2^Y = 0.438), precluding any further analysis (Table S10).

## 3. Discussion

### 3.1. Relationships among Key Aroma Compounds, Odor-Active Compounds, Aromatic Series, and Consumer-Liking Scores

Twenty-six of the 100 compounds emerged as the key compounds relating to predicted aroma consumer likings (Table S9). Of the 26, only 13 compounds (OAVs > 1; Tables S3 and S4) contributed to both consumer liking and aroma intensity. Also, as in popular cultivars [10], octanal (VIP = 1.60) and β-ionone (VIP = 1.98) determined the overall aroma quality of unfamiliar cultivars. The other 13 volatiles, despite remaining non-aromatic (OAVs < 1), influenced consumer liking by interacting with other aroma compounds [6,10]. On the other hand, it was interesting to note that the esters responsible for rich fruity notes failed to influence consumer liking. Generally, volatiles affect the overall aroma in two ways: directly, by being active, and indirectly, via blending and the resulting interactions (e.g., additive, etc.) [6,10,34,35]. It is possible that esters followed neither of these two phenomena, and hence, were ineffective in influencing consumer liking. This imposes a major challenge in determining the relative contributions made by a large number of diverse chemicals to flavor and liking [35].

**Figure 6:**
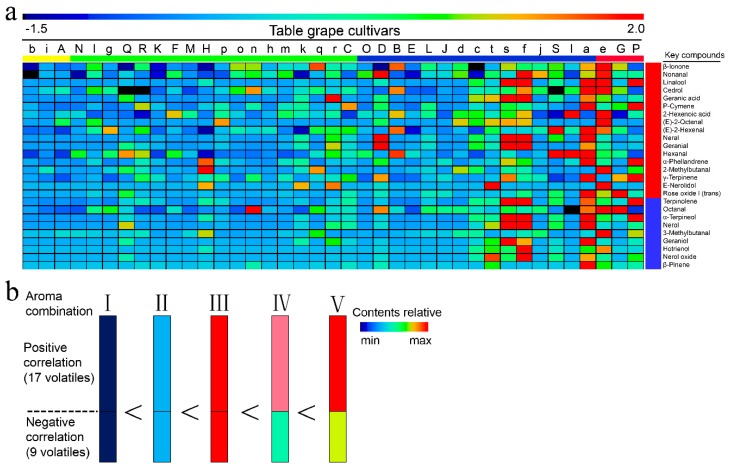
(**a**) Correlation between heatmap of key aroma compounds and the predicted aroma consumer liking (variable importance in projection (VIP) > 1). Upper and lower cases indicate table-grape cultivars, as listed in Figure 1 and Figure 4. The yellow, green, blue, and red colors correspond to poor, average, good, and excellent aroma liking scores, respectively. The key compounds are listed in the right panel. Data are given as means. (**b**) The model of aroma combinations for all table grapes. In each combination, the upper part indicates the content of volatiles for positive relationships, and the lower part represents the content of volatiles for negative relationships.

Between the two table-grape groups (unfamiliar and popular), the cultivars A, b, and i (see Figure 5 for cultivars corresponding to lower-case letters) showed the lowest scores, whereas G, P, and e cultivars showed the highest scores (Table S8). The scores of the remaining cultivars ranged from 3.5 to 4.5. Based on these scores, the aromatics were classified into four groups (Figure 6a): excellent (scores > 4.5), good (4.0–4.5), average (3.5–4.0), and poor aroma (<3.5). Analysis of key volatiles in these groups (Figure 6a) revealed that the contents were overall low, resulting in low scores, especially for b, i, and A. However, as their contents increased only to a certain extent, the liking scores increased as shown by the average aroma cultivars (3.5–4.0). Any further increase, as that which occurred in good-aroma cultivars, did not increase the liking scores. On the other hand, an increase in key volatiles of cultivars such as s, f, a, and e showed both positive and negative relationships with the liking scores. These patterns emphasized that consumer liking depended on the combination of positive and negative relationships, offering additional perspective to the phenomenon of aroma perception. These combinations, termed in this study as aroma combinations (AC; Figure 6b), are as follows, based on the contents of key compounds: (I) < (II) < (IV) < (V) < (III). This grouping revealed that a high content of certain volatiles in some cultivars lowered their liking score. This is why the grape berries with only an appropriate AC, and not the highest aroma (Figure 6b), demonstrated high liking scores, which eventually led to consumer preference.

Akin to popular cultivars [10], the unfamiliar cultivars also followed the five primary aromatic series (Figure S1a,b), further corroborating that all table grapes possess such aromas. Just like the primary series, the seven secondary series, including apple, citrus, orange, rose, geranium, violet, and honey, endowed the cultivars with specific aroma characteristics. For instance, although the cultivars G and P both showed high scores, they belonged to different groups, and differed in aroma OAV profiles (Figure 2i). Similarly, of the popular cultivars, consumers preferred Shine Muscat (e) with a balsamic aroma (primary series) [10]. Interestingly, even though the balsamic aroma was much lower in all unfamiliar cultivars, some of them such as P and G obtained high liking scores. This could be attributable to their unique inherent aroma influencing the consumer likings. Furthermore, the cultivar P had rich citrus, grape, rose, lavender, and marshmallow aromas, whereas G showed high values for orange, lemon, and floral aromas (Figure 2i). These results demonstrated that secondary aromatic series could enhance liking scores by generating diverse flavor combinations, enriched with enticing aromas. Nevertheless, studies showed that consumer liking primarily depended on the specific aromatic series contributed by volatiles [30,31,36,37,38]. Our study also supports this conclusion; however, unlike the other studies, we developed a model to acquire aromatic series values and liking scores (Table 1; Table S8). 

### 3.2. The Screening of Unfamiliar Cultivars

The flavor, and eventually, the marketability of consumer-preferred table grapes can be improved by identifying novel aromas with high liking scores. This study revealed that the liking scores for G (Xiangyue) and P (Heikuixiang) were the greatest (Figure 5a and Table S8), even much greater than the most popular cultivar, Shine Muscat [10]. Although both cultivars showed high scores, they differed in sensory attributes (Figure 3). Cultivar P exhibited high values for citrus, grape, rose, lavender, and marshmallow aromas, while G showed strong orange, lemon, and flower aromas. These aromatic differences yield a wide array of flavors, which could be used by the table-grape breeders to devise a blueprint for improving flavor, and eventually, a paradigm of consumer acceptance of table grapes worldwide.

### 3.3. Comprehensive Description of Aroma Characteristics in Table Grapes

**Figure 7:**
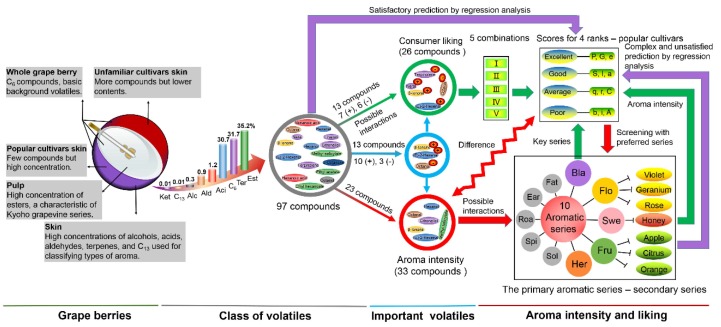
Schematic diagram showing the overall aroma characteristics of table grapes from a chemical perspective. The chemical types and primary aromatic series are represented by the first three letters of their names. Column heights indicate the average content of each chemical type, and for convenient viewing, the column heights of five volatiles (alcohols, acids, aldehydes, ketones, and C_13_-norisoprenoids) are amplified ten times their original height.

Numerous (100) volatiles were identified in both table-grape groups (Figure 7). Among these, C_6_ compounds occurred as the basic background volatiles. The aroma contents of pulp and skin depended mainly on the levels of esters and terpenes, respectively. The esters constituted the signature aromas of the “Kyoho grapevine series” berries [10], whereas terpenes formed the basis for *classifying Vitis vinifera* cultivars [10]. Esters (35.20%) dominated among the eight volatiles, followed by terpenes (31.69%) and C_6_ compounds (30.67%; Table S11). About one-fourth of volatiles (26) emerged as the key volatiles, wherein nine volatiles related negatively, and 17 related positively to liking scores, with β-Ionone occurring as the most important compound. Thirteen of these key compounds influenced consumer liking via aroma interactions. Finally, based on the liking scores, the table grapes were classified into four groups, in which about one-third of volatiles (33) directly contributed to aroma intensity. The overall aroma liking depended upon the integration of primary (balsamic) and secondary aromatic series. Accordingly, Xiangyue and Heikuxiang emerged as the acceptable cultivars.

## 4. Materials and Methods

### 4.1. Materials

Nineteen unfamiliar table-grape cultivars were collected from a single vineyard (31° N, 74° E, Shanghai, China) from July to September 2015. These cultivars included small plantings of low-yielding landraces, and local or new varieties; hence, they were unknown to most consumers. Their characteristics and other pertinent details are described in the Supplementary Materials (Table S12). All cultivars in this study were grown uniformly in terms of age of the vines (five years old), training and trellising, pruning, irrigation, soil type, fertilization, etc. Standard cultural practices, as in popular cultivars [10], were adopted to maintain healthy vines. Such uniform management practices yielded berries specific to individual cultivars. The sampling protocol, processing, and analysis were identical to that of traditional/popular cultivars [10], except for the reduction in sample size in this study. Briefly, for each cultivar, six healthy clusters from three vines, taking into account the number of berries per cluster, and the exposure of clusters to sun and shade (approximately three kilograms), were randomly picked at their respective appropriate commercial harvest dates. After sampling, the clusters were transported to the laboratory at 4 °C. Three clusters were processed for standard chemical analysis, and the remaining clusters were immediately frozen at −80 °C until the aroma analysis was performed.

### 4.2. Chemicals

Some pertinent chemicals included methanol (HPLC grade; Merck, Darmstadt, Germany) and chemical standards (details are provided in the Supplementary Materials). Others included SPME fibres of 50/30 µm divinylbenzene/carboxen/polydimethylsiloxane (DVB/CAR/PDMS; Supelco, Bellefonte, PA, USA).

### 4.3. Sample Preparation

Thirty-six berries from different positions (top, middle, and bottom) of the three clusters were randomly collected as a replicate, thoroughly rinsed, and dried with filter paper. Then, the berries were squeezed to release the juice using a homogenizer, centrifuged at 10,000× *g* for 10 min at 4 °C, and filtered using cellulose paper. The juice was analyzed for total soluble solids (TSS), titratable acidity (TA), and pH analysis. 

We followed the methodology of our previous study [10] to prepare samples, and to perform quantitative and qualitative analyses of volatiles. Briefly, after thawing (4 °C), the berries of each batch (200 g) were manually and carefully peeled, de-stemmed, and deseeded. Subsequently, the skins were immediately immersed into bottles containing extraction solutions (pH 3.2, 3 g/L of tartaric acid, 50 mg/L of Vc). The skins were stirred in this buffer solution for 24 h at 20 °C. Deseeded pulps were homogenized for 1 min after the addition of 50 mg/L Vc. Thereafter, the extractions of skin and pulp juice in triplicate were used for volatile analysis.

### 4.4. SPME-GC-MS

The sample (6 mL), NaCl (1.5 g), and 2-octanol internal standard solution (5 μL, 155 mg/L) were transferred to 20-mL glass vials (1/β = 0.5) [10], before the vials were capped. The volatiles were equilibrated for 10 min at 50 °C, and then extracted for 30 min with SPME fiber. After the extraction, the fiber was immediately inserted into the GC injection port to desorb volatiles at 260 °C for 3 min in splitless mode. Compounds were identified by matching their mass spectra and retention times with that of the standard NIST 2011 library and the retention times reported in the literature. In the case of compounds for which standards were not available in the library, their retention times were compared with those reported in the literature. Compounds were quantified using calibration curves. For compounds without calibration curves, semi-quantitative determinations were made using the internal standards. The desorbed volatiles were separated in an Agilent 7890 GC (Santa Clara, CA, USA) equipped with HP-INNOWAX (30 m × 0.25 mm i.d., 0.25 µm film thickness; J & W Scientific, Folsom, CA, USA), and coupled with an Agilent 5975 MS. The temperature program entailed 40 °C for 5 min; thereafter, it was increased to 240 °C at 5 °C min^−1^, and then ramped up at 20 °C min^−1^ to 260 °C, which was held for 5 min. Because of the similarities in cultural practices and the quantitative and qualitative analyses, it was possible to perform a comparative analysis of the data between the popular and unfamiliar cultivars. 

### 4.5. Calculation of the Aromatic Series Values

Descriptors and thresholds for the volatiles were obtained (Table S7) from previous reports. Since some compounds had one or several aromas (odor descriptors), the aroma compounds with similar descriptors were grouped into one aromatic series. Such classification yielded the grouping of all compounds into ten primary aromatic series: herbaceous, floral, fruity, sweet, spicy, roasty, fatty, earthy, balsamic, and solvent. Among them, three (floral, fruity, and sweet) were rather broad and diverse. Consequently, these were further divided into very specific aromas (i.e., 14, 13, and 5 secondary aromatic series, respectively). Compounds that belonged to these three primary series also followed the secondary aromatic series. For example (Table S5), ethyl propionate had banana, apple, and strawberry aromas. Based on the above principle, it simultaneously represented the fruity primary series, and the banana, apple, and strawberry secondary series.

### 4.6. Sensory Analysis and Consumer-Liking Scores

In our previous study [10], we identified aroma compounds in 20 popular table-grape cultivars. The consumer preference for these cultivars was determined using market feedback and aroma evaluation based on a 1-to-5-point scale. In the presented study, such analysis was not possible with the unfamiliar cultivars because of their low yields, small plantings, and the fact that they were new to the market. To tackle this problem, an orthogonal partial least squares (OPLS) was performed to extract linear relationships from two data blocks, the volatile contents (X) and liking scores (Y), from the popular cultivars [10], and then, this information was utilized to predict the liking scores of unfamiliar cultivars.

### 4.7. Statistical Analysis

One-way analysis of variance (ANOVA) and hierarchical cluster analysis (HCA) were performed using the SPSS 19 software; the orthogonal projection to latent structures (OPLS) and principal component analysis (PCA) were performed using the SIMCA 14.1 software; the heatmaps were visualized with the MultiExperiment Viewer version 4.8 software. The remainder of the figures were made using the Origin 9.1 software. PCA, HCA, and OPLS were weighted using unit variance (UV) scaling. 

### 4.8. Chemometric Analysis

While performing PCA, Hotelling’s T2 (99%) was used to determine and eliminate strong outliers. For the OPLS model, the number of latent components was determined using a seven-fold cross-validation technique. Its performance is described by the following statistical terms: *R*^2^X, *R*^2^Y, *Q*^2^Y, the coefficient of correlation (*R*^2^), the root-mean-square error of cross-validation (RMSECV), and root-mean-square error of estimation (RMSEE) [38,39]. The *R*^2^ and RMSEE values measured the suitability of the model; *R*^2^Y estimated its accuracy, whereas *Q*^2^Y estimated predictive ability. Other statistics used to evaluate the predictive ability of the model included the root-mean-square error of prediction (RMSEP) and the root-mean-square error of cross-validation (RMSECV). RMSEP was calculated from an independent dataset (external validation), whereas RMSECV was calculated from samples within the dataset [38,39].

Generally, *Q*^2^Y > 0.5 was considered as good, and *Q*^2^Y > 0.9 as excellent. Furthermore, the differences between *R*^2^X and *Q*^2^X were not to be too large, preferably not exceeding 0.2–0.3. Because of the limited number of training sets, the prediction ability of the model could not be evaluated through external validation (root-mean-square error of prediction, RMSEP). Hence, an alternative measure of predicting the model was pursued by summarizing the cross-validation residuals of the observations in the workset. The resulting prediction measure was termed RMSECV, and was used to evaluate the prediction ability of the model. In addition, a permutation test (*n* = 200) on the Y block was performed to safely overcome casualty or over fitting. The variable importance in projection (VIP) plot summarized the importance of the variables in explaining X and in correlating with Y, yielding the identification of important volatiles. Since the average of squared VIP scores was equal to 1, a greater-than-one rule was generally used as a criterion for variable selection [38]. 

## 5. Conclusions

We used the natural variation in aroma volatiles of unfamiliar table-grape cultivars to improve consumer liking. A total of 100 compounds were identified. Of these, 26 compounds were selected as the key compounds, and their correlation with consumer liking resulted in the formation of five aroma/liking combinations. On the other hand, 33 compounds contributed to aroma and impacted the aroma intensity. Five primary aromatic series distinguished all table grapes, whereas fruity, floral, and sweet aromas contributed to 14, 13, and 5 secondary aromatic series, respectively. The overall liking of the cultivars ensued from the combination of certain preferred primary aromatic and secondary aromatic series. Xiangyue and Heikuixiang emerged as the preferred table grapes. These findings permit table-grape breeders to devise a blueprint for improving flavor by focusing on a specific number of chemicals. 

## Figures and Tables

**Table 1:** Summary of the results of orthogonal partial least squares (OPLS) model developed for pulp juice, skin, and whole-grape berries.

Attribute	No. of Latent Variable	*R*^2^X	*R*^2^Y	*Q*^2^Y	*R* ^2^	RMSEE	RMSEcv
Pulp juice	1 + 6 + 0	0.693	0.919	0.799	0.9188	0.2016	0.2952
Skin	1 + 5 + 0	0.643	0.865	0.725	0.8645	0.2601	0.3484
Grape berries	1 + 7 + 0	0.753	0.942	0.834	0.9419	0.17215	0.2681

*R*^2^X—the ratio of the variance of the X data set; *R*^2^Y—the ratio of the variance of the Y data set; *Q*^2^Y—the cross-validated *R*^2^Y; *R*^2^—the coefficient of correlation; RMSEE—root-mean-square error of estimation; RMSEcv—root-mean-square error of cross-validation. *R*^2^Y indicates the accuracy of model, whereas *Q*^2^Y indicates its predictability.

## Supplementary Materials

The following are available online, Figure S1: Primary aromatic series values of unfamiliar table grape. Figure S2: Hierarchical cluster analysis (HCA) of active secondary aromatic series for unfamiliar table grape. Figure S3: Overview and diagnostic of models established by compounds contents. Figure S4: Overview and diagnostic of models established by aromatic series values. Table S1: Concentrations (μg/kg) of volatile compounds determined in the pulp juice of unfamiliar table grape cultivars. Table S2: Concentrations (μg/kg) of volatile compounds determined in the skin of unfamiliar table grape cultivars. Table S3: Odor activity values (OAVs) of active volatile compounds determined in the pulp juice of unfamiliar cultivars table grapes. Table S4: Odor activity values (OAVs) of active volatile compounds determined in the skin of unfamiliar cultivars table grapes. Table S5: Each class of volatile compounds as measured in the pulp juice, skin and whole grape berries in popular and unfamiliar cultivars table grapes. Table S6: Terpenes volatile compounds as measured in skin for unfamiliar and popular cultivars table grapes. Table S7: Chemical standards, retention index (RI), odour descriptors, odorant series, odour threshold (μg/L) of the studied compounds. Table S8: Significant differences of predicted aroma consumer liking scores by ANOVA analysis followed by Duncan’s test (*p* = 0.05). Table S9: The key aroma compounds correlated with predicted aroma consumer liking selected by variable selection procedure (VIP > 1) using OPLS regression model, their VIP scores and coefficients. Table S10: The results of OPLS models established by primary aromatic series values. Table S11: The average concentrations and percentage of different types compounds in 39 table grapes. Table S12: Information for unfamiliar table grape cultivars in this study.

## Abbreviations

SPME	solid-phase microextraction
GC-MS	gas chromatography/mass spectrometry
DVB/CAR/PDMS	divinylbenzene/carboxen/polydimethylsiloxane
TSS	total soluble solids
TA	titratable acidity
ANOVA	one-way analysis of variance
HCA	hierarchical cluster analysis
OPLS	orthogonal projection to latent structures
PCA	principal component analysis
UV	unit variance
RMSECV	root-mean-square error of cross-validation
RMSEE	root-mean-square error of estimation
RMSEP	root-mean-square error of prediction
VIP	variable importance in projection
OAVs	the odor activity values
AC	aroma combinations
