# Supplementary material for: In-Depth Aroma and Sensory Profiling of Unfamiliar Table-Grape Cultivars
Source: Molecules. 2018 Jul 12;23(7):1703. doi: 10.3390/molecules23071703 (PMC6100037; doi:10.3390/molecules23071703)
Supplement: Supplementary file 1 [file molecules-23-01703-s001.pdf]

In-Depth Aroma and Sensory Profiling of Unfamiliar Table-Grape Cultivars

Yusen Wu [1], Wenwen Zhang [1], Shuyan Duan [1], Shiren Song [1], Wenping Xu [1], Caixi Zhang [1], Bhaskar Bondada [2,*], Chao Ma [1,*], Shiping Wang [1,3,*]

[1] Department of Plant Science, School of Agriculture and Biology, Shanghai Jiao Tong University, 800 Dongchuan Road, Minhang District, Shanghai 200240, China
[2] Wine Science Center, Washington State University, Richland, WA 99354, USA
[3] Institute of Agro-food Science and Technology/Key Laboratory of Agro-products, Processing Technology of Shandong, Shandong Academy of Agricultural Sciences, Jinan 250100, People's Republic of China
* Correspondence: e-mail address: bbondada@wsu.edu (B. Bondada), chaoma2015@sjtu.edu.cn (C.M.), fruit@sjtu.edu.cn (S.W); Tel.: +86-151-211-70281

Supplementary Materials and Methods.

The chemical standards were purchased as follows: (*Z*)-3-hexenal, 2-octanol, hexanal, (*E*)-2-hexenal, geranic acid, phellandrene, β-myrcene, β-damascenone, D-limonene, citronellol, benzyl alcohol, neral, α-terpineol, geranial, geraniol, rose oxide II (cis), rose oxide I (trans), phenylethyl alcohol from Sigma (St. Louis, MO, USA); octanoic acid, pentanal, octanal, nonanal, benzaldehyde, 3-methylbutanal, hexanoic acid, (*Z*)-3-hexenol, 1-octen-3-ol, heptanol, ethyl acetate, ethyl butyrate, ethyl isobutyrate, butyl acetate, ethyl pentanoate, methyl salicylate, hexyl acetate, (*E*)-2-hexenoic acid, P-cymene, terpinolene, linalool, 4-terpineol, geranyl acetone from Dr. Ehrenstorfer (Germany); β-ionone from Fluka (Buchs, Switzerland); ethyl hexanoate from Nu-chek (USA); octanol, hexanol, (*E*)-2-hexenol from Chem Service (USA), and n-alkanes (C_7-C_{27}) from Supelco (Bellefonte, PA).

Figures

Figure S1. Primary aromatic series values of unfamiliar table grape. The primary aromatic series values for pulp juice (**a**) and skin (**b**). Data are represented as mean ± SD (n = 3). Capital letters refer to the unfamiliar cultivars table grape as listed in **Figure 1**.

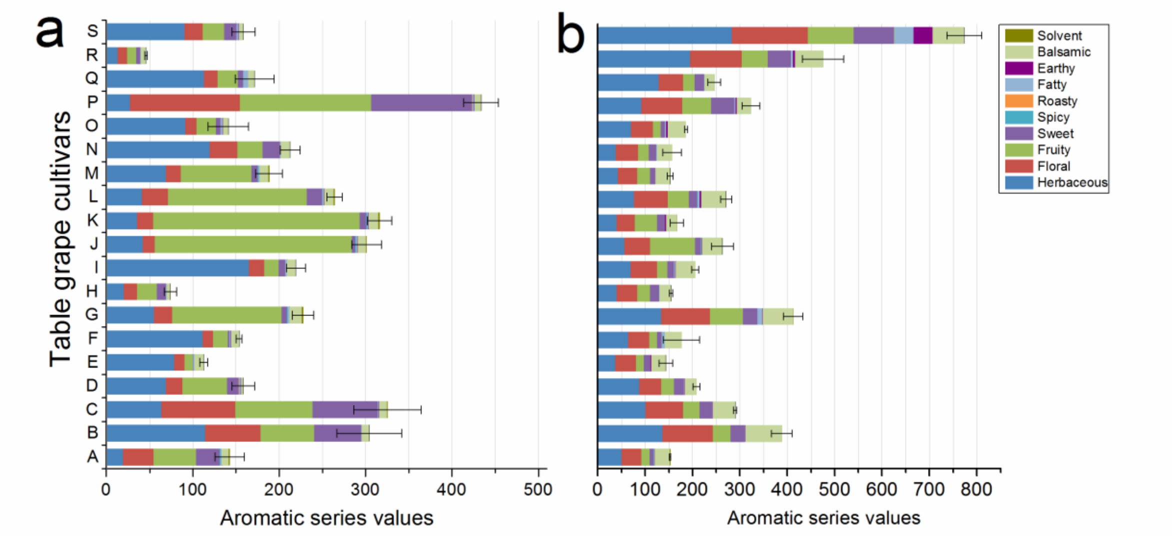

Figure S2. Hierarchical cluster analysis (HCA) of active secondary aromatic series for unfamiliar table grape. (**a**) Pulp juice samples are divided into five clusters: groups p1, p2, p3, p4, and p5. (**b**) Skin samples are divided into six clusters: groups s1, s2, s3, s4, s5 and s6. (**c**) Whole grape berry samples are divided into five clusters: groups g1, g2, g3, g4, and g5. Capital letters refer to the unfamiliar cultivars table grape as listed in **Figure 1**.

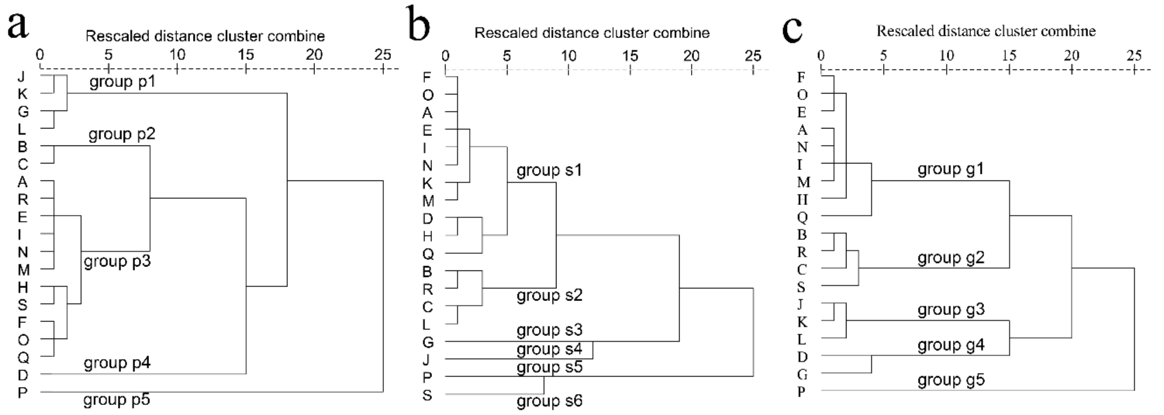

Figure S3. Overview and diagnostic of models established by compounds contents. Overview principal component analysis (PCA) score plots obtained from the pulp juice (**a**), skin (**c**) and whole grape berries (**g**). (**e**) Overview principal component analysis (PCA) score plots obtained from skin which excluding the strong outliers from the skin (**c**). Hotelling's T^2 obtained from the pulp juice (**b**), skin (**d**) and whole grape berries (**h**). (**f**) Hotelling's T^2 obtained from the skin which excluding the strong outliers from the skin (**d**). In this study, Hotelling's T2 (99%) was used to find the strong outlier, which outside the 99% tolerance region. The strong outlier did not fit the model well and should be excluded from the OPLS analysis. (**i**) Permutation test performed with 200 rounds of random permutations of the Y variable performed on the training set samples (whole grape berries). Small letters refer to the popular cultivars table grape as listed in **Figure 4**.

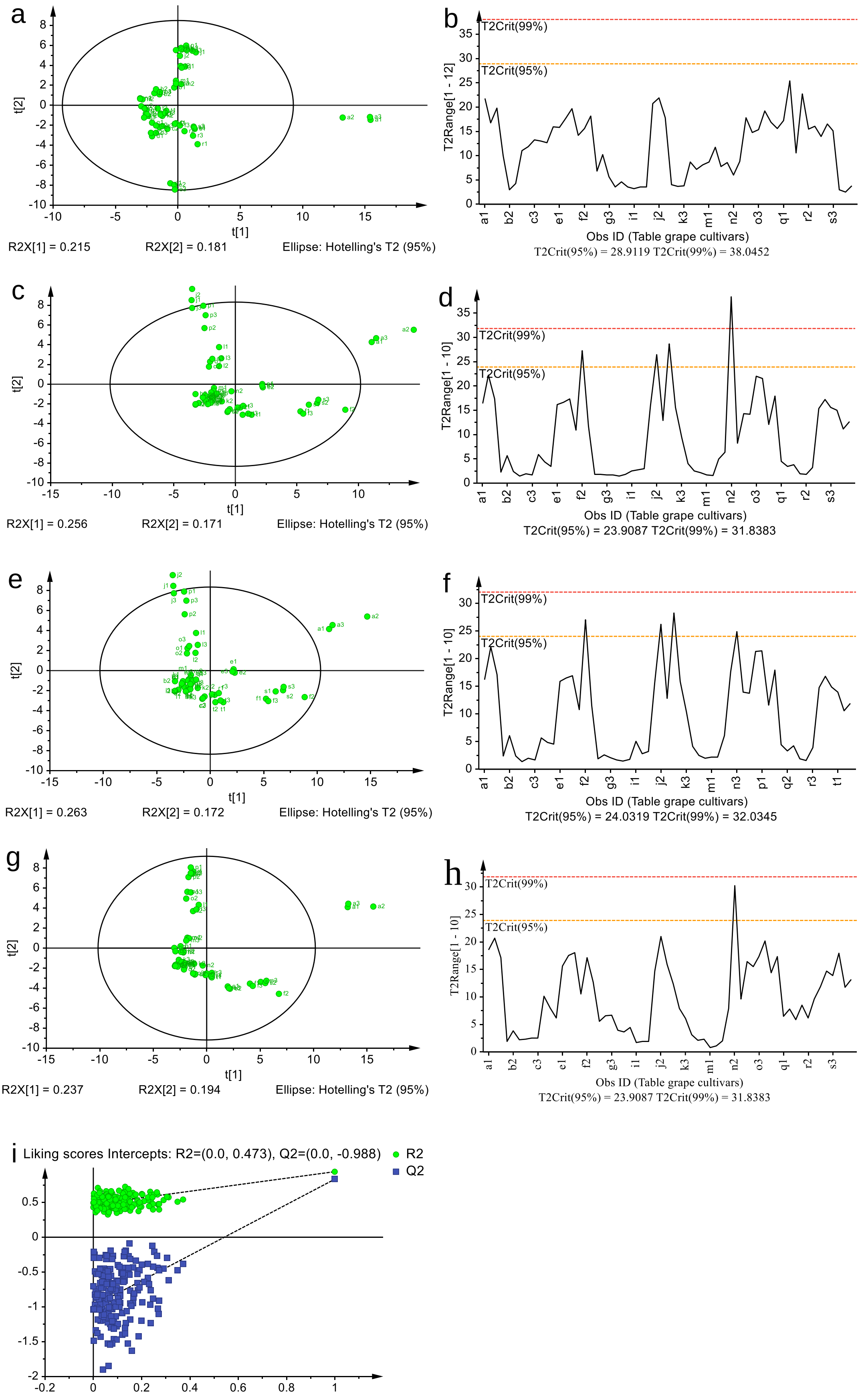
a
t[2]
t[1]
R2X[1] = 0.215
R2X[2] = 0.181
Ellipse: Hotelling's T2 (95%)
b
T2Crit(99%)
T2Crit(95%)
T2Range[1 - 12]
Obs ID (Table grape cultivars)
T2Crit(95%) = 28.9119 T2Crit(99%) = 38.0452
c
t[2]
t[1]
R2X[1] = 0.256
R2X[2] = 0.171
Ellipse: Hotelling's T2 (95%)
d
T2Crit(99%)
T2Crit(95%)
T2Range[1 - 10]
Obs ID (Table grape cultivars)
T2Crit(95%) = 23.9087 T2Crit(99%) = 31.8383
e
t[2]
t[1]
R2X[1] = 0.263
R2X[2] = 0.172
Ellipse: Hotelling's T2 (95%)
f
T2Crit(99%)
T2Crit(95%)
T2Range[1 - 10]
Obs ID (Table grape cultivars)
T2Crit(95%) = 24.0319 T2Crit(99%) = 32.0345
g
t[2]
t[1]
R2X[1] = 0.237
R2X[2] = 0.194
Ellipse: Hotelling's T2 (95%)
h
T2Crit(99%)
T2Crit(95%)
T2Range[1 - 10]
Obs ID (Table grape cultivars)
T2Crit(95%) = 23.9087 T2Crit(99%) = 31.8383
i
Liking scores Intercepts: R2=(0.0, 0.473), Q2=(0.0, -0.988)
R2
Q2

Figure S4. Overview and diagnostic of models established by aromatic series values. Overview principal component analysis (PCA) score plot (**a**) and Hotelling's T^2 (**b**) obtained from the whole grape berries based on primary aromatic series. In this study, Hotelling's T2 (99%) was used to find the strong outlier, which outside the 99% tolerance region. The strong outlier did not fit the model well and should be excluded from the OPLS analysis. Small letters refer to the popular cultivars table grape as listed in **Figure 4**.

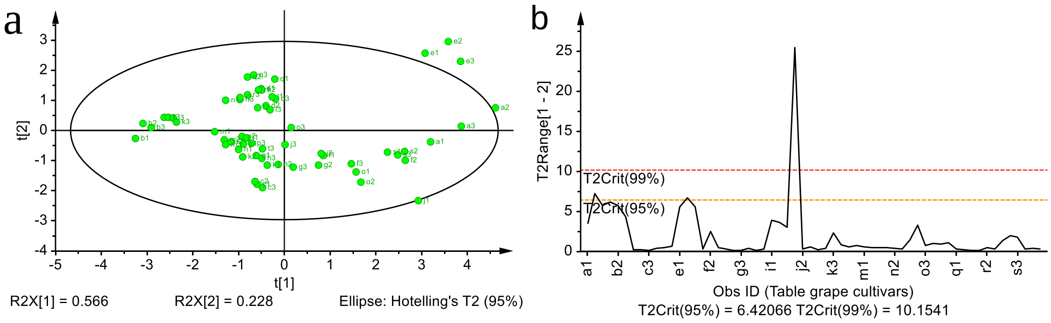

Table S1. Concentrations (μg/kg) of volatile compounds determined in the pulp juice of unfamiliar table grape cultivars. Data are means (*n* = 3). The capital letters refer to the unfamiliar cultivars table grape listed in **Figure 1**. The aroma compounds were listed on the left of the concentration arrays, and the colour scale was shown at the bottom. The higher concentration for each compound was presented in red; otherwise, green was used; - indicated that the compound was not detected.* indicated that semi-quantitative determinations were made using the internal standards without any calibration curves, and the other compounds were quantified using calibration curves.

Compounds	Cultivars																		
	A	B	C	D	E	F	G	H	I	J	K	L	M	N	O	P	Q	R	S
A) C6 alcohols																			
Hexanal	28.08	325.84	145.49	153.07	264.13	361.46	152.65	25.45	576.01	103.44	56.41	111.99	201.77	409.97	304.17	18.99	422.27	19.93	330.71
(*Z*)-3-Hexenal	-	0.59	0.28	-	0.16	0.16	0.18	-	0.18	-	0.06	-	0.06	0.29	0.22	-	0.07	-	0.43
(*E*)-2-Hexenal	49.09	528.56	321.29	195.62	142.18	208.02	158.36	5.90	375.54	65.53	126.51	78.67	148.89	247.50	103.97	27.45	69.87	13.29	111.34
Hexanol	13.82	87.32	33.55	76.98	135.33	163.45	20.66	17.14	169.50	73.07	111.70	80.81	99.02	113.23	165.61	18.71	115.03	5.77	62.98
(*E*)-3-Hexenol	-	-	-	-	0.43	14.95	1.27	-	8.05	-	0.65	-	-	1.48	0.59	3.86	-	-	-
(*Z*)-3-Hexenol	3.21	2.87	5.88	27.49	58.94	41.63	5.21	0.30	121.99	6.86	14.41	41.16	30.66	24.23	5.63	12.53	1.33	-	1.61
(*E*)-2-Hexenol	22.54	92.17	42.37	47.83	117.24	146.90	18.65	5.14	377.15	101.40	316.91	39.60	162.33	222.23	162.87	22.18	96.04	19.98	127.15
SubTOTAL	116.74	1037.35	548.86	500.99	718.42	936.56	356.98	53.93	1628.41	350.29	626.66	352.22	642.74	1018.92	743.05	103.72	704.60	58.98	634.21
%	2.82	94.00	28.75	53.72	90.88	88.43	6.88	26.63	84.85	8.13	14.34	16.12	16.01	90.82	92.32	10.95	87.30	56.53	85.18
(B) Alcohols																			
Isopropanol *	0.74	0.14	0.37	-	-	-	-	0.04	-	4.57	0.22	0.42	1.38	-	-	0.18	-	1.16	0.03
2-Methyl-3-butene-2-ol *	0.51	-	1.39	-	-	-	2.26	0.12	-	-	-	-	-	-	-	-	-	0.29	0.05
3-methyl-2-Butanol *	0.06	-	-	-	-	-	-	-	-	0.04	-	-	0.07	-	-	-	-	0.27	0.12
Pentanol *	-	0.05	-	-	-	-	-	-	-	-	-	-	-	-	-	-	-	-	-
1-Octen-3-ol	0.22	0.22	0.13	0.71	0.44	0.60	0.35	0.14	0.34	0.35	0.93	0.36	0.18	0.23	0.32	0.21	0.22	0.14	0.29
Heptanol	0.26	0.31	0.34	0.25	0.36	0.22	0.29	0.13	0.28	0.26	0.67	0.39	0.69	0.32	0.29	0.24	-	-	-
2-Ethyl hexanol *	1.04	1.36	1.20	2.01	2.51	3.63	1.28	1.80	1.29	1.38	1.44	1.79	1.72	1.43	1.20	1.91	2.10	0.97	1.44
Octanol	-	0.29	0.38	1.16	-	0.65	-	0.09	-	0.25	0.68	0.28	0.32	-	0.37	0.65	0.19	-	0.26
Nonanol	0.27	0.16	-	0.32	0.11	0.21	0.20	0.06	0.16	0.34	0.81	0.11	0.30	0.17	0.23	0.39	0.25	0.33	0.10
Benzyl alcohol	-	-	-	-	-	-	-	-	-	-	-	0.26	-	-	-	-	-	-	-
Phenylethyl alcohol	1.33	-	0.29	0.23	0.12	0.03	0.57	-	0.10	0.12	1.60	2.63	0.10	0.05	-	0.67	-	-	-
SubTOTAL	4.42	2.51	4.09	4.69	3.53	5.34	4.94	2.37	2.16	7.31	6.36	6.23	4.76	2.19	2.41	4.24	2.76	3.16	2.29
%	0.11	0.23	0.21	0.50	0.46	0.50	0.10	1.17	0.11	0.17	0.15	0.29	0.12	0.20	0.30	0.45	0.34	3.03	0.31
(C) Esters																			
Ethyl acetate	3974.42	4.99	1300.74	1.79	16.69	9.45	4618.79	18.66	48.24	3693.99	3488.11	1612.91	3242.22	54.13	20.01	244.07	16.84	17.03	0.80
Ethyl Propionate *	0.20	-	0.25	-	-	-	1.27	-	-	0.86	2.30	1.04	0.80	-	-	0.06	-	-	-
Ethyl isobutyrate	-	-	-	-	-	-	-	-	-	0.10	0.20	0.07	-	-	-	-	-	-	-
Propyl acetate	0.45	-	0.08	-	-	-	0.26	-	-	0.32	0.09	0.08	0.18	-	-	0.01	-	-	-
Ethyl butyrate	2.72	-	2.60	-	-	-	65.94	-	-	116.49	121.70	103.64	41.89	-	-	14.34	-	-	-
Ethyl 2-methylbutanoate *	0.31	-	0.05	-	-	-	1.57	-	-	4.11	3.73	0.49	0.37	-	-	0.02	-	-	-
Ethyl 3-methylbutanoate *	-	-	-	-	-	-	0.05	-	-	0.03	0.07	-	-	-	-	-	-	-	-
Butyl acetate	0.12	0.05	0.05	0.15	0.05	0.05	0.07	0.02	0.11	0.24	0.05	0.05	0.27	-	0.02	0.15	-	-	-

Ethyl pentanoate	-	-	-	-	-	-	2.74	-	-	4.03	3.99	2.10	1.97	-	-	0.05	-	-	-
(*Z*)-2-Butenoic acid ethyl ester *	-	-	3.14	-	-	-	20.07	-	-	13.42	3.19	9.70	5.06	-	0.09	0.28	-	0.03	-
Methyl hexanoate *	-	-	-	-	-	-	-	-	-	0.08	0.10	-	-	-	-	-	0.05	-	-
Ethyl hexanoate	8.70	1.97	3.03	0.75	2.32	1.20	24.06	1.32	2.82	50.89	58.34	24.50	18.42	2.51	2.01	7.53	2.28	1.83	1.61
Hexyl acetate	0.20	0.01	-	-	0.15	0.03	0.06	-	0.19	1.15	0.15	0.17	0.16	0.09	0.08	0.42	-	-	-
(*E*)-2-Hexenyl acetate *	-	-	-	-	-	-	-	-	-	0.60	-	-	-	-	-	0.23	-	-	-
(*Z*)-3-Hexenyl acetate *	-	-	-	-	-	0.02	-	-	0.23	0.34	-	0.54	-	-	-	0.24	-	-	-
Ethyl heptanoate *	-	-	-	-	-	-	0.66	-	-	-	1.03	0.89	-	-	-	-	-	-	-
2-Hexenoic acid ethyl ester *	0.84	-	0.14	-	-	-	1.03	-	-	5.42	4.42	1.83	1.67	-	-	0.33	-	-	-
Ethyl octanoate *	-	-	-	-	-	-	0.70	-	-	0.39	0.62	-	0.19	-	-	-	-	-	-
Ethyl 3-hydroxybutyrate *	0.70	-	-	-	-	-	2.14	-	-	1.62	0.98	-	1.92	-	-	-	-	-	-
Methyl salicylate	-	-	0.25	0.10	0.49	0.06	0.18	0.06	0.01	0.62	0.82	1.53	0.64	0.18	-	0.15	-	-	-
SubTOTAL	3988.67	7.02	1310.34	2.79	19.70	10.81	4739.58	20.05	51.59	3894.70	3689.90	1759.53	3315.77	56.91	22.21	267.87	19.17	18.90	2.41
%	96.21	0.64	68.63	0.30	2.49	1.02	91.31	9.90	2.69	90.40	84.46	80.55	82.61	5.07	2.76	28.28	2.37	18.11	0.32
(D) Acids																			
Hexanoic acid	2.86	2.34	0.82	0.60	1.34	0.96	0.61	0.28	4.88	2.46	1.46	1.00	1.55	3.23	0.96	0.51	2.75	-	0.08
2-Hexenoic acid	18.11	44.36	5.84	18.89	14.58	62.28	2.79	2.24	154.16	15.36	10.21	23.46	23.69	17.61	9.42	18.78	9.52	-	-
Octanoic acid	0.42	0.24	0.13	0.53	0.18	0.47	3.34	0.10	1.24	0.30	0.19	0.27	0.40	0.68	1.02	1.28	-	-	-
Nonanoic acid *	0.80	0.54	0.62	0.83	0.15	0.26	0.18	0.23	0.19	0.27	0.17	0.06	0.15	0.24	0.19	0.21	-	-	-
Decanoic acid *	0.20	0.10	-	0.10	0.03	0.05	0.04	0.04	0.04	0.06	0.05	0.02	0.04	0.05	0.04	0.04	-	-	-
SubTOTAL	22.39	47.57	7.41	20.96	16.28	64.02	6.96	2.89	160.51	18.44	12.07	24.81	25.83	21.81	11.63	20.82	12.27	-	0.08
%	0.01	0.04	0.00	0.02	0.02	0.06	0.00	0.01	8.36	0.00	0.00	0.01	0.01	0.02	0.01	0.02	0.02	0.00	0.00
(E)Aldehydes																			
2-Methylbutanal *	-	-	-	-	0.04	0.02	-	0.46	-	-	-	-	-	-	-	0.10	0.24	0.12	-
3-Methylbutanal	-	-	-	-	0.21	0.04	-	1.12	-	-	-	0.11	-	0.41	0.09	1.40	0.36	0.56	-
Pentanal	-	-	-	-	-	-	-	-	-	-	-	-	-	-	-	-	0.05	-	0.06
Heptanal	-	-	-	0.09	0.14	-	0.05	0.05	-	0.05	0.07	0.13	0.11	0.12	0.18	0.12	0.33	0.06	0.13
Octanal	0.29	0.18	0.37	1.03	0.32	0.57	0.38	0.45	-	0.45	0.33	0.31	0.36	0.30	0.38	0.46	0.69	0.20	0.29
Nonanal	7.14	5.67	7.97	18.35	5.79	12.54	6.25	6.86	5.76	8.35	6.20	6.88	9.11	5.85	12.56	11.12	9.91	3.99	6.51
Decanal*	-	-	-	-	-	-	-	0.04	-	-	-	-	-	-	0.14	0.11	0.47	-	0.19
Benzaldehyde	0.51	0.96	7.87	8.54	4.03	2.47	0.55	0.45	9.69	1.14	1.48	5.45	0.83	2.91	0.18	1.03	0.10	1.87	12.78
Phenylacetaldehyde *	0.64	0.05	0.70	-	0.14	0.65	-	0.44	0.09	0.57	0.28	1.98	0.69	0.29	0.07	1.90	-	-	-
SubTOTAL	8.58	6.85	16.90	28.02	10.68	16.29	7.24	9.87	15.54	10.56	8.36	14.86	11.11	9.88	13.59	16.25	12.16	6.80	19.96
%	0.21	0.62	0.89	3.00	1.35	1.54	0.14	4.87	0.81	0.25	0.19	0.68	0.28	0.88	1.69	1.72	1.51	6.52	2.68
(F) Terpenes																			
α-Pinene	-	-	-	2.69	0.11	-	0.40	-	0.11	-	0.09	-	-	-	-	-	-	-	-
β-Pinene	-	-	-	1.09	-	-	0.33	0.19	0.18	-	0.07	-	-	-	-	0.97	-	-	0.22
α-Phellandrene	0.18	-	0.64	0.43	0.08	0.12	0.30	0.18	0.08	0.08	0.51	0.48	0.08	0.09	0.07	1.10	0.18	0.92	0.09
β-Myrcene	-	-	-	0.84	0.56	0.69	0.49	2.36	0.56	0.52	0.58	1.14	0.52	0.60	0.50	1.75	1.24	0.71	1.48
D-Limonene	-	-	-	188.09	9.14	17.44	37.31	40.47	8.46	5.26	6.98	5.75	5.31	4.35	5.58	12.98	9.60	0.54	5.01
Eucalyptol *	-	-	-	-	-	-	-	-	-	-	0.20	-	-	-	-	-	-	-	-
β-Phellandrene	-	-	-	1.64	-	-	-	-	-	-	-	0.11	-	-	-	0.20	-	0.56	-

γ-Terpinene*	-	-	1.05	3.78	0.30	0.40	0.96	0.26	0.18	1.74	0.68	0.27	0.65	0.30	0.17	3.34	-	0.79	-
β-cis-Ocimene*	-	-	-	0.50	-	-	-	-	-	-	-	-	-	-	-	1.00	-	-	0.43
P-Cymene	-	-	0.57	0.71	-	0.04	0.26	0.15	-	-	0.24	0.57	0.01	-	-	2.59	0.05	0.41	-
Terpinolene	-	-	0.75	0.81	0.05	0.19	0.46	0.75	0.05	0.05	0.76	0.82	0.17	0.06	0.05	8.59	0.22	0.47	0.57
Rose oxide II (cis)	-	-	-	0.22	-	-	-	0.03	-	-	-	0.26	-	-	-	0.30	0.27	-	0.14
Rose oxide I (trans)	-	-	0.07	0.12	-	-	0.37	0.07	-	0.14	-	0.15	-	-	-	0.12	0.42	0.08	-
cis-Linalool oxide*	-	-	-	-	-	-	-	0.27	-	-	-	-	-	-	-	-	-	-	0.06
Nerol oxide*	-	-	-	0.00	-	-	-	-	-	-	-	-	-	-	-	0.97	-	-	-
Linalool	-	-	0.33	60.93	-	-	12.02	52.65	1.46	-	-	0.23	-	-	-	328.30	1.36	-	50.37
4-Terpineol	0.21	-	2.36	-	-	-	4.30	-	-	0.97	1.26	1.69	0.60	-	-	8.49	0.08	1.50	-
Hotrienol*	-	0.15	0.17	0.26	-	-	0.29	0.75	-	-	-	-	-	-	-	-	-	-	0.38
Menthol*	-	0.05	0.15	0.04	-	0.02	-	0.48	-	0.42	-	0.32	-	-	-	-	0.19	-	-
Neral	-	-	-	1.92	-	-	-	-	-	-	-	-	-	-	-	-	1.44	-	-
α-Terpineol	0.48	0.44	1.43	2.22	0.33	0.36	8.19	4.50	1.22	0.14	1.29	1.62	0.50	0.41	0.37	53.98	1.24	0.19	3.00
Geranial	0.22	-	0.46	5.08	0.54	0.18	0.34	0.17	0.61	0.07	1.12	0.26	0.07	0.24	0.07	1.03	1.83	0.10	0.91
Citronellol	0.72	-	1.72	4.48	1.27	0.67	2.55	1.12	2.28	0.75	2.36	2.73	1.55	0.86	0.73	3.48	2.21	1.58	3.67
Nerol	-	-	3.31	14.28	2.20	1.25	1.33	3.58	2.72	1.41	3.81	4.13	1.41	1.62	1.36	16.05	13.08	2.72	7.70
Geraniol	-	-	5.60	25.04	1.87	1.25	1.77	4.27	1.50	1.41	3.97	4.88	1.45	1.62	1.36	45.05	16.41	4.82	10.37
Cedrol	0.79	0.90	0.95	0.28	0.64	1.11	0.70	0.11	0.80	0.53	0.68	0.37	0.48	0.90	0.65	0.30	0.23	0.29	0.23
Geranic acid	1.26	0.49	1.87	63.46	4.37	1.66	1.77	0.53	40.47	13.21	0.60	0.57	0.54	0.62	0.52	42.81	5.51	0.74	0.65
SubTOTAL	3.86	2.03	21.43	378.91	21.49	25.38	74.15	112.88	60.68	26.70	25.19	26.36	13.33	11.67	11.43	533.40	55.55	16.40	85.26
%	0.09	0.18	1.12	40.16	2.73	2.40	1.43	55.70	3.16	0.62	0.58	1.21	0.34	1.04	1.42	56.32	6.88	15.72	11.45
(G) C13-Norisoprenoids																			
β-Damascenone	0.05	0.11	0.15	0.00	-	0.00	0.01	0.00	0.01	0.01	0.01	0.03	0.01	0.04	0.01	0.12	0.01	0.01	0.01
Geranyl acetone	0.24	-	-	0.25	0.07	0.20	0.15	0.09	0.07	0.30	0.20	0.23	0.08	0.25	0.17	0.29	0.10	0.02	0.08
β-Ionone	0.05	0.06	0.06	0.02	0.07	0.06	0.09	0.03	0.07	0.06	0.07	0.07	0.06	0.07	0.04	0.05	0.05	0.04	0.03
SubTOTAL	0.35	0.17	0.21	0.27	0.14	0.26	0.25	0.12	0.15	0.37	0.28	0.33	0.16	0.37	0.22	0.46	0.16	0.07	0.12
%	0.01	0.02	0.01	0.03	0.02	0.02	0.00	0.06	0.01	0.01	0.01	0.02	0.00	0.03	0.03	0.05	0.02	0.07	0.02
(H) Ketones																			
6-Methyl-5-hepten-2-one*	0.83	0.11	0.10	0.40	0.07	0.41	0.12	0.48	0.05	-	0.24	0.09	-	0.19	0.34	0.34	0.44	0.02	0.24
SubTOTAL	0.83	0.11	0.10	0.40	0.07	0.41	0.12	0.48	0.05	-	0.24	0.09	-	0.19	0.34	0.34	0.44	0.02	0.24
%	0.02	0.01	0.01	0.04	0.01	0.04	0.00	0.24	0.00	0.00	0.01	0.00	0.00	0.02	0.04	0.04	0.05	0.02	0.03
TOTAL	4145.84	1103.62	1909.35	932.67	790.54	1059.07	5190.45	202.54	1919.10	4308.29	4369.06	2184.45	4013.94	1121.94	804.88	947.15	807.10	104.33	744.57

relative

row min row max

Table S2. Concentrations (μg/kg) of volatile compounds determined in the skin of unfamiliar table grape cultivars. Data are means (*n* = 3). The capital letters refer to the unfamiliar cultivars table grape listed in Figure 1. The aroma compounds were listed on the left of the concentration arrays, and the colour scale was shown at the bottom. The higher concentration for each compound was presented in red; otherwise, green was used; - indicated that the compound was not detected. * indicated that semi-quantitative determinations were made using the internal standards without any calibration curves, and the other compounds were quantified using calibration curves.

Compounds	Cultivars																		
	A	B	C	D	E	F	G	H	I	J	K	L	M	N	O	P	Q	R	S
(A) C6 compounds																			
Hexanal	104.24	360.44	218.63	103.72	80.27	125.28	255.23	74.15	159.67	95.01	79.10	157.54	86.05	77.32	147.31	132.77	198.99	538.25	543.52
(*Z*)-3-Hexenal	0.23	1.04	0.96	0.46	0.26	0.86	1.87	0.32	0.79	0.25	0.26	0.84	0.56	0.48	1.18	0.63	0.67	1.30	3.11
(*E*)-2-Hexenal	247.38	653.84	599.95	280.86	133.85	341.94	646.97	184.72	337.38	360.43	245.97	432.37	251.34	245.44	439.83	365.45	425.00	909.92	1542.44
Hexanol	13.39	84.87	15.60	36.03	234.28	45.44	22.65	178.48	66.67	55.45	77.08	44.12	28.17	67.84	56.57	20.00	38.52	283.70	618.05
(*E*)-3-Hexenol	1.74	0.91	-	-	2.98	0.76	15.34	3.23	0.35	6.03	1.41	-	-	0.82	0.75	2.44	-	-	-
(*Z*)-3-Hexenol	-	1.42	4.17	4.93	27.95	4.35	7.35	0.42	11.89	2.87	6.35	23.66	13.05	5.43	2.33	3.42	0.34	-	-
(*E*)-2-Hexenol	14.09	100.79	31.28	47.90	324.15	58.50	29.63	46.36	26.96	66.15	149.28	21.45	82.48	64.87	73.83	27.84	32.03	282.23	779.38
SubTOTAL	386.84	1225.82	901.98	489.06	808.40	591.03	1007.41	499.91	613.60	597.15	566.01	697.06	471.21	468.71	736.28	561.56	703.46	2031.03	3508.65
%	23.21	90.46	50.18	19.53	79.16	84.22	23.83	51.08	72.24	20.18	49.32	54.28	41.36	81.02	90.58	22.09	19.40	86.08	58.12
(B) Alcohols																			
Butanol *	-	-	-	-	-	-	-	-	-	-	-	-	-	-	-	0.03	-	-	-
1-Octen-3-ol	0.68	0.66	0.56	0.91	0.90	0.47	1.04	0.20	0.69	0.75	1.65	0.88	0.40	0.63	0.33	0.53	0.48	1.43	2.47
Heptanol	0.61	0.65	0.98	0.27	0.30	0.18	0.68	0.41	0.27	0.66	0.82	0.84	0.30	0.24	0.33	-	0.09	0.67	0.62
2-Ethyl hexanol *	1.06	1.76	2.80	3.85	1.62	2.23	3.67	1.85	3.96	2.40	1.68	4.61	2.47	1.50	2.14	1.80	1.77	3.80	4.94
Octanol	0.87	0.80	1.21	0.33	0.43	0.31	1.22	0.15	0.58	0.82	1.79	2.09	0.53	0.42	0.42	0.66	0.10	1.78	1.03
Nonanol	0.54	0.58	1.13	0.17	0.28	0.11	1.43	0.29	0.34	0.32	2.09	1.31	0.26	0.30	0.14	0.54	0.10	0.93	1.19
Benzyl alcohol	-	-	-	0.09	-	-	-	-	-	0.12	-	-	0.11	-	-	0.33	-	-	0.63
Phenylethyl alcohol	-	-	-	0.16	-	-	1.25	-	-	0.41	4.68	5.51	0.10	0.09	0.07	1.36	0.07	0.18	0.79
SubTOTAL	3.76	4.46	6.68	5.79	3.53	3.31	9.29	2.91	5.83	5.49	12.72	15.25	4.18	3.17	3.42	5.26	2.61	8.80	11.66
%	0.23	0.33	0.37	0.23	0.35	0.47	0.22	0.29	0.69	0.19	1.11	1.19	0.37	0.56	0.43	0.21	0.08	0.37	0.19
(C) Esters																			
Ethyl acetate	1145.70	6.29	148.29	4.27	70.07	-	2268.06	212.23	11.50	2156.87	438.73	200.47	548.35	18.19	1.56	22.80	18.87	8.57	145.89
Ethyl Propionate *	-	-	-	-	-	-	0.16	-	-	0.15	0.11	-	0.08	-	-	-	-	-	-
Propyl acetate	0.03	-	-	-	-	-	-	-	-	-	-	-	-	-	-	-	-	-	-
Ethyl butyrate	-	-	-	-	-	-	5.53	-	-	21.99	5.68	8.23	4.71	-	-	1.24	-	-	-
Ethyl 2-methylbutanoate *	0.14	-	-	-	-	-	-	-	-	2.11	0.72	-	0.34	-	-	-	-	-	-
Butyl acetate	0.04	-	0.10	0.03	0.04	0.06	0.09	0.04	0.03	0.06	-	0.16	0.09	-	0.03	0.02	-	-	-
Ethyl pentanoate	-	-	-	-	-	-	-	-	-	1.91	0.11	0.16	-	-	-	-	-	-	-
(*Z*)-2-Butenoic acid ethyl ester *	0.97	-	-	-	-	-	4.31	-	-	2.92	0.43	1.20	1.54	-	-	-	-	-	-
Ethyl hexanoate	4.68	3.13	4.48	-	1.24	2.06	12.71	-	2.06	29.47	11.32	10.75	6.08	1.72	2.57	5.81	-	-	-
Hexyl acetate	0.02	0.08	-	-	4.57	-	-	2.59	0.08	0.25	-	-	-	0.22	-	-	0.14	-	0.81
(*Z*)-3-Hexenoic acid ethyl	0.15	-	-	-	-	-	0.26	-	-	0.36	-	-	0.21	-	-	-	-	-	-

ester*																			
(Z)-3-Hexenyl acetate *	-	-	-	-	-	-	-	-	-	-	-	0.41	-	-	-	-	-	-	-
Ethyl heptanoate	-	-	-	-	-	-	0.38	-	-	-	0.35	0.17	-	-	-	-	-	-	-
(Z)-2-Hexenyl acetate *	0.49	-	-	-	3.55	-	-	0.94	-	1.54	-	-	0.48	0.20	-	-	-	-	0.91
2-Hexenoic acid ethyl ester *	0.38	-	-	-	-	-	-	-	-	4.47	1.88	1.13	1.01	-	-	0.72	-	-	-
Ethyl octanoate *	0.45	-	-	-	-	-	0.72	-	-	0.72	0.55	0.51	0.08	-	-	-	-	-	-
Ethyl 3-hydroxybutyrate *	-	-	-	-	-	-	0.18	-	-	0.08	-	-	0.16	-	-	-	-	-	-
Benzoic acid ethyl ester *	-	-	-	-	-	-	-	-	-	0.31	-	1.09	-	-	-	0.13	-	-	-
Methyl salicylate	0.94	0.27	8.85	0.25	0.73	0.26	-	0.12	0.24	8.22	1.98	8.05	3.62	0.64	0.50	0.62	-	-	-
SubTOTAL	1153.98	9.76	161.72	4.55	80.21	2.38	2292.40	215.91	13.91	2231.43	461.85	232.33	566.76	20.97	4.66	31.34	19.01	8.57	147.61
%	69.24	0.59	9.00	0.19	7.85	0.34	54.22	22.06	1.64	75.41	40.25	18.10	49.74	3.62	0.57	1.22	0.52	0.36	2.45
(D) Acids																			
Hexanoic acid	-	1.87	-	-	0.66	0.58	-	-	2.05	1.07	0.22	0.35	0.76	0.65	0.64	-	-	-	-
2-Hexenoic acid	30.56	63.91	-	26.26	25.33	45.28	-	20.35	73.99	-	17.57	46.10	44.78	30.18	44.27	45.28	8.97	-	-
Octanoic acid	-	1.17	-	-	0.34	-	-	-	0.71	1.52	0.44	2.19	1.37	1.00	1.66	-	2.73	2.34	7.08
Nonanoic acid *	-	0.41	-	0.28	-	0.06	0.28	0.12	0.18	-	-	-	-	-	0.25	-	0.06	-	-
Decanoic acid *	-	0.07	-	0.03	0.01	0.03	0.03	0.02	0.02	-	0.03	0.04	0.03	-	-	0.02	-	-	-
SubTOTAL	30.56	67.44	0.00	26.57	26.34	45.95	0.31	20.50	76.95	2.59	18.26	48.68	46.95	31.83	46.82	45.30	11.76	2.34	7.08
%	1.83	4.98	0.00	1.06	2.58	6.55	0.01	2.09	9.11	0.09	1.60	3.80	4.12	5.50	5.76	1.76	0.36	0.15	0.12
(E) Aldehydes																			
2-Methylbutanal *	-	-	-	-	-	-	-	0.21	-	-	-	0.08	-	-	-	0.18	-	-	-
3-Methylbutanal	0.46	0.10	-	-	-	-	-	0.57	-	0.29	-	0.29	0.40	-	-	0.29	-	-	-
Pentanal	0.69	1.97	1.55	1.18	-	1.49	3.28	-	0.59	0.60	-	1.88	1.74	0.43	0.75	1.70	1.28	4.01	-
Heptanal	-	-	-	0.12	0.06	0.11	0.26	-	-	-	0.08	0.26	0.09	0.09	0.14	0.10	0.12	0.25	0.31
Octanal	0.26	0.69	0.53	0.43	0.19	0.32	1.44	0.21	0.29	0.57	0.18	0.78	0.20	0.22	0.30	-	0.13	0.32	0.36
(Z)-2-Heptenal *	-	-	-	0.21	-	0.25	-	-	0.13	-	-	-	0.03	-	0.07	-	0.37	-	0.20
Nonanal	3.91	3.84	1.60	2.40	2.40	2.60	7.54	2.09	3.03	4.10	2.26	5.62	1.44	1.69	3.75	2.76	2.18	6.30	9.74
(E)-2-Octenal *	0.12	-	-	0.12	-	-	-	-	-	-	-	-	-	-	-	-	-	-	-
Decanal	-	-	-	-	-	-	0.13	-	-	-	-	-	0.04	0.06	0.07	0.06	-	-	0.24
Benzaldehyde	6.73	6.57	33.60	12.93	17.97	2.49	14.38	1.66	7.03	27.52	15.24	111.71	5.62	14.98	3.32	9.90	5.98	11.37	171.72
(E)-2-Nonenal *	-	-	-	-	-	-	-	-	0.08	-	-	0.21	-	-	0.20	0.14	-	0.27	3.00
(E,Z)-2,6-Nonadienal *	-	-	-	-	-	0.12	0.11	-	-	-	-	-	-	-	-	-	-	-	-
Phenylacetaldehyde	0.93	0.39	-	-	0.11	0.32	0.64	0.96	-	1.57	-	2.96	1.17	0.44	-	1.29	-	-	-
2,5-Dimethylbenzaldehyde *	0.46	0.32	-	0.23	-	-	-	0.20	0.58	-	-	-	-	-	-	-	-	-	-
SubTOTAL	13.56	13.87	37.29	17.63	20.73	7.70	27.77	5.89	11.72	34.66	17.76	123.79	10.72	17.90	8.59	16.43	10.06	22.52	185.57
%	0.81	1.02	2.07	0.72	2.03	1.10	0.66	0.61	1.41	1.17	1.55	9.66	0.95	3.14	1.09	0.65	0.28	0.95	3.08
(F) Terpenes																			
α-Pinene	-	-	-	-	-	-	0.38	0.61	-	-	-	-	-	-	-	-	-	-	-
β-Pinene	-	-	1.01	0.26	-	-	0.50	0.18	-	0.25	0.23	0.23	-	-	-	0.67	-	0.55	-
α-Phellandrene	-	0.35	-	1.50	-	-	-	3.01	0.75	0.87	-	-	-	-	0.14	2.70	-	0.15	-
β-Myrcene	-	-	4.84	2.09	1.01	1.66	6.25	-	1.49	-	1.47	2.01	1.54	1.44	-	-	3.10	-	6.35

α-Terpinene *	0.07	-	2.70	0.38	-	-	1.36	0.79	0.07	0.18	-	0.25	0.11	-	0.05	3.45	0.83	1.69	0.40
D-Limonene	-	15.53	23.89	2.09	5.53	17.41	115.60	61.11	23.07	43.05	23.34	25.31	17.58	24.35	8.86	13.37	2.17	-	2.98
β-trans-Ocimene *	-	-	-	0.56	-	-	-	0.91	-	-	-	-	-	-	-	0.58	0.69	-	2.16
γ-Terpinene *	0.22	0.42	2.36	0.45	0.15	0.46	3.13	1.84	0.38	-	1.16	1.09	0.74	0.59	0.18	3.92	0.88	2.09	-
β-cis-Ocimene *	-	-	-	0.95	-	-	-	-	-	-	-	-	-	-	-	-	0.28	-	-
P-Cymene	0.14	0.26	1.00	0.32	0.13	0.21	0.92	0.38	0.19	0.26	0.25	0.35	0.17	0.17	0.19	0.90	0.22	0.96	0.35
Terpinolene	0.54	-	4.40	1.54	-	0.44	2.79	4.43	0.83	0.66	1.67	1.74	0.51	-	0.60	14.96	2.72	3.72	6.42
Rose oxide I (trans)	-	-	0.23	0.13	-	-	0.92	-	-	0.09	-	0.13	-	-	-	0.17	0.18	-	0.52
cis-Linalool oxide *	-	-	-	-	-	-	-	-	-	-	-	-	-	-	-	0.27	-	-	-
Nerol oxide *	-	-	0.12	0.62	-	-	-	0.17	-	-	-	-	-	-	-	1.26	1.06	-	1.56
Citronellal *	-	-	0.13	-	-	-	-	-	-	-	-	-	-	-	-	-	0.05	-	-
Linalool	0.42	-	13.40	36.40	0.39	-	24.56	46.21	7.92	0.24	0.63	1.45	0.13	-	0.25	148.63	31.78	2.27	90.26
4-Terpineol	0.41	-	3.60	-	-	-	4.39	0.41	-	0.98	0.84	3.15	0.61	-	-	7.45	0.39	4.42	0.42
Hotrienol *	-	-	-	0.36	-	-	0.33	0.68	-	-	-	-	-	-	-	0.87		0.11	0.92
Menthol *	0.08	0.29	0.15	0.10	0.14	0.11	0.25	-	0.20	-	0.23	-	-	-	0.45	-	0.23	0.24	0.40
Neral	-	-	7.34	42.96	1.10	0.27	-	0.96	0.89	0.38	2.28	7.67	-	0.75	-	4.96	-	-	20.08
α-Terpineol	0.09	-	21.45	14.25	-	-	16.13	26.68	2.52	-	3.65	3.44	-	-	-	80.63	19.27	6.09	38.16
Geranial	0.54	0.92	11.16	73.09	2.54	-	4.28	2.35	1.21	0.50	3.06	13.06	0.27	1.09	-	7.15	21.26	10.85	-
Citronellol	2.54	-	74.65	11.36	-	-	256.35	0.97	0.92	4.66	1.63	12.51	0.55	-	-	31.40	14.66	4.86	68.03
Myrtenol *	-	-	0.66	-	-	-	-	-	-	-	0.08	-	-	-	-	0.24	-	-	-
Nerol	3.55	-	138.94	78.36	0.93	-	33.19	10.18	1.01	1.74	10.84	13.04	0.35	-	-	150.69	308.88	4.48	217.20
Geraniol	9.12	-	162.85	87.16	2.75	-	34.17	30.94	7.04	2.55	9.43	11.95	2.72	0.02	-	233.68	351.94	14.79	171.52
E-Nerolidol *	-	-	-	0.03	-	-	-	0.16	-	-	-	-	-	-	-	-	-	-	-
Cedrol	1.85	3.44	1.89	1.18	0.71	1.11	2.98	1.60	2.52	1.32	0.71	0.95	0.66	0.64	1.11	0.98	-	0.16	-
Eugenol *	0.35	-	0.51	-	0.18	-	0.00	-	0.03	0.21	0.29	0.63	0.16	0.13	-	0.34	-	-	0.97
Geranic acid	57.33	12.71	209.98	1598.46	65.91	28.70	380.19	36.60	74.41	27.44	8.44	66.19	12.20	5.67	-	1187.88	2116.66	225.08	1543.21
SubTOTAL	77.26	33.92	688.35	1954.61	81.46	50.38	889.07	232.89	125.47	85.38	70.24	165.15	38.29	34.86	11.82	1901.07	2878.49	282.50	2171.92
%	4.64	2.50	38.28	78.11	7.98	7.18	21.03	23.80	14.79	2.94	6.12	12.88	3.39	6.03	1.45	74.02	79.35	11.99	35.98
(G) C13-Norisoprenoids																			
β-Damascenone	0.01	0.06	0.05	0.04	0.02	0.02	0.05	0.02	0.03	0.02	0.03	0.03	0.02	0.03	0.02	0.05	0.03	0.10	0.14
Geranyl acetone	0.12	0.19	0.29	0.20	0.10	0.16	0.34	0.13	0.16	0.10	0.10	0.19	0.15	0.11	0.17	0.25	-	0.29	0.49
β-Ionone	0.23	0.54	0.33	0.17	0.22	0.24	0.44	0.17	0.29	0.29	0.16	0.37	0.22	0.22	0.27	0.21	0.14	0.41	0.47
SubTOTAL	0.36	0.78	0.66	0.42	0.34	0.42	0.83	0.32	0.47	0.42	0.29	0.59	0.38	0.36	0.45	0.51	0.17	0.80	1.09
%	0.02	0.06	0.04	0.02	0.03	0.06	0.02	0.03	0.06	0.02	0.03	0.05	0.03	0.06	0.06	0.02	0.00	0.03	0.02
(H) Ketones																			
2-Octanone *	0.24	0.56	0.73	0.26	0.15	0.25	0.43	0.28	0.22	0.25	0.19	0.31	0.21	0.18	0.31	-	-	0.39	-
6-Methyl-5-hepten-2-one *	-	0.30	0.19	3.21	-	0.30	0.49	-	0.40	-	0.16	0.30	0.22	0.16	0.23	0.98	0.48	0.73	3.00
SubTOTAL	0.24	0.86	0.92	3.46	0.15	0.55	0.92	0.28	0.62	0.25	0.35	0.62	0.44	0.34	0.54	0.98	0.48	1.12	3.00
%	0.01	0.06	0.05	0.14	0.02	0.08	0.02	0.03	0.07	0.01	0.03	0.05	0.04	0.06	0.07	0.04	0.01	0.05	0.05
TOTAL	1666.56	1356.91	1797.61	2502.08	1021.16	701.71	4227.99	978.61	848.58	2957.35	1147.47	1283.47	1138.92	578.13	812.59	2562.45	3626.04	2357.68	6036.58

relative

row min row max

Table S3. Odor activity values (OAVs) of active volatile compounds determined in the pulp juice of unfamiliar cultivars table grapes. Data are means (*n* = 3). - indicated that the compound was not detected. Gray represents the value equal to or greater than 1. The capital letters refer to the unfamiliar cultivars table grape listed in **Figure 1.**

	Cultivars																		
**Compounds**	**A**	**B**	**C**	**D**	**E**	**F**	**G**	**H**	**I**	**J**	**K**	**L**	**M**	**N**	**O**	**P**	**Q**	**R**	**S**
**(A) C_6 alcohols**																			
Hexanal	6.24	72.41	32.33	34.02	58.70	80.32	33.92	5.66	128.00	22.99	12.54	24.89	44.84	91.10	67.59	4.22	93.84	4.43	73.49
(*Z*)-3-Hexenal	-	2.37	1.11	-	0.66	0.66	0.70	-	0.72	-	0.24	-	0.25	1.17	0.89	-	0.27	-	1.70
(*E*)-2-Hexenal	2.89	31.09	18.90	11.51	8.36	12.24	9.32	0.35	22.09	3.85	7.44	4.63	8.76	14.56	6.12	1.61	4.11	0.78	6.55
(*Z*)-3-Hexenol	0.05	0.04	0.08	0.39	0.84	0.59	0.07	<0.01	1.74	0.10	0.21	0.59	0.44	0.35	0.08	0.18	0.02	-	0.02
(*E*)-2-Hexenol	0.23	0.92	0.42	0.48	1.17	1.47	0.19	0.05	3.77	1.01	3.17	0.40	1.62	2.22	1.63	0.22	0.96	0.20	1.27
SubTOTAL	9.40	106.83	52.84	46.39	69.73	95.28	44.20	6.06	156.32	27.95	23.59	30.50	55.91	109.40	76.31	6.24	99.19	5.41	83.04
%	13.91	59.56	34.11	45.15	75.93	76.77	23.67	14.99	84.64	10.43	8.52	14.85	37.46	71.52	71.57	3.25	74.47	20.80	72.39
**(C) Esters**																			
Ethyl isobutyrate	-	-	-	-	-	-	-	-	-	1.01	1.99	0.69	-	-	-	-	-	-	-
Ethyl butyrate	2.72	-	2.60	-	-	-	65.94	-	-	116.49	121.70	103.64	41.89	-	-	14.34	-	-	-
Ethyl 2-methylbutanoate	3.43	-	0.60	-	-	-	17.20	-	-	45.19	41.01	5.37	4.11	-	-	0.26	-	-	-
Ethyl pentanoate	-	-	-	-	-	-	1.83	-	-	2.69	2.66	1.40	1.31	-	-	0.03	-	-	-
(*Z*)-2-Butenoic acid ethyl ester	-	-	0.23	-	-	-	1.48	-	-	0.99	0.23	0.71	0.37	-	<0.01	0.02	-	<0.01	-
Ethyl hexanoate	8.70	1.97	3.03	0.75	2.32	1.20	24.06	1.32	2.82	50.89	58.34	24.50	18.42	2.51	2.01	7.53	2.28	1.83	1.61
**SubTOTAL**	14.85	1.97	6.46	0.75	2.32	1.20	110.51	1.32	2.82	217.26	225.94	136.30	66.10	2.51	2.02	22.18	2.28	1.83	1.61
**%**	21.98	1.10	4.17	0.73	2.53	0.97	59.18	3.27	1.53	81.06	81.55	66.35	44.28	1.64	1.89	11.58	1.71	7.05	1.40
**(E)Aldehydes**																			
3-Methylbutanal	-	-	-	-	1.07	0.18	-	5.61	-	-	-	0.55	-	2.05	0.43	7.01	1.80	2.79	-
Octanal	0.41	0.25	0.53	1.48	0.46	0.82	0.55	0.64	-	0.65	0.48	0.45	0.51	0.43	0.54	0.65	0.99	0.29	0.42
Nonanal	7.14	5.67	7.97	18.35	5.79	12.54	6.25	6.86	5.76	8.35	6.20	6.88	9.11	5.85	12.56	11.12	9.91	3.99	6.51
Decanal	-	-	-	-	-	-	-	0.42	-	-	-	-	-	-	1.40	1.11	4.71	-	1.85
**SubTOTAL**	7.55	5.92	8.49	19.83	7.32	13.53	6.80	13.53	5.76	9.00	6.68	7.88	9.63	8.33	14.94	19.89	17.41	7.07	8.77
**%**	11.18	3.30	5.48	19.30	7.97	10.90	3.64	33.47	3.12	3.36	2.41	3.83	6.45	5.45	14.01	10.38	13.07	27.15	7.65
**(F) Terpenes**																			
D-Limonene	-	-	-	18.81	0.91	1.74	3.73	4.05	0.85	0.53	0.70	0.57	0.53	0.44	0.56	1.30	0.96	0.05	0.50
Rose oxide II (cis)	-	-	1.50	1.61	0.11	0.38	0.91	1.50	0.11	0.10	1.52	1.65	0.34	0.12	0.10	17.18	0.44	0.93	1.14
Linalool	-	-	0.05	10.16	-	-	2.00	8.78	0.24	-	-	0.04	-	-	-	54.72	0.23	-	8.39
Geraniol	-	-	0.14	0.63	0.05	0.03	0.04	0.11	0.04	0.04	0.10	0.12	0.04	0.04	0.03	1.13	0.41	0.12	0.26
Cedrol	1.58	1.80	1.90	0.56	1.29	2.22	1.41	0.21	1.61	1.06	1.37	0.75	0.96	1.80	1.29	0.60	0.46	0.59	0.45
**SubTOTAL**	1.58	1.80	3.60	31.76	2.36	4.37	8.10	14.64	2.85	1.72	3.68	3.13	1.87	2.39	1.98	74.92	2.50	1.69	10.75
**%**	2.34	1.00	2.32	30.91	2.57	3.52	4.34	36.21	1.54	0.64	1.33	1.52	1.25	1.57	1.86	39.11	1.88	6.49	9.37

**(G) C13-Norisoprenoids**																			
β-Damascenone	26.68	54.26	74.49	1.72	-	0.87	4.02	0.81	7.09	3.45	7.01	17.10	6.70	19.83	4.97	60.69	4.48	4.37	5.57
β-Ionone	7.50	8.61	9.04	2.30	10.11	8.86	13.10	4.07	9.85	8.65	10.15	10.52	9.07	10.50	6.40	7.65	7.34	5.65	4.96
**SubTOTAL**	34.18	62.87	83.53	4.02	10.11	9.73	17.13	4.88	16.95	12.11	17.16	27.62	15.76	30.33	11.37	68.34	11.81	10.02	10.53
**%**	50.59	35.05	53.91	3.91	11.01	7.84	9.17	12.07	9.18	4.52	6.19	13.44	10.56	19.83	10.67	35.67	8.87	38.50	9.18
**TOTAL**	67.56	179.38	154.93	102.75	91.84	124.11	186.74	40.42	184.69	268.03	277.05	205.42	149.27	152.97	106.61	191.57	133.20	26.02	114.70

Table S4. Odor activity values (OAVs) of active volatile compounds determined in the skin of unfamiliar cultivars table grapes. Data are means (*n* = 3). - indicated that the compound was not detected. Gray represents the value equal to or greater than 1. The capital letters refer to the the unfamiliar cultivars table grape listed in **Figure 1.**

	**Cultivars**																		
**Compounds**	**A**	**B**	**C**	**D**	**E**	**F**	**G**	**H**	**I**	**J**	**K**	**L**	**M**	**N**	**O**	**P**	**Q**	**R**	**S**
**(A) C6 compounds**																			
Hexanal	23.16	80.10	48.58	23.05	17.84	27.84	56.72	16.48	35.48	21.11	17.58	35.01	19.12	17.18	32.74	29.51	44.22	119.61	120.78
(*Z*)-3-Hexenal	0.90	4.17	3.85	1.83	1.05	3.46	7.49	1.29	3.16	1.01	1.05	3.36	2.25	1.92	4.72	2.54	2.66	5.21	12.42
(*E*)-2-Hexenal	14.55	38.46	35.29	16.52	7.87	20.11	38.06	10.87	19.85	21.20	14.47	25.43	14.78	14.44	25.87	21.50	25.00	53.52	90.73
Hexanol	0.03	0.17	0.03	0.07	0.47	0.09	0.05	0.36	0.13	0.11	0.15	0.09	0.06	0.14	0.11	0.04	0.08	0.57	1.24
(*E*)-2-Hexenol	0.14	1.01	0.31	0.48	3.24	0.58	0.30	0.46	0.27	0.66	1.49	0.21	0.82	0.65	0.74	0.28	0.32	2.82	7.79
**SubTOTAL**	38.79	123.91	88.07	41.95	30.47	52.09	102.60	29.46	58.89	44.10	34.74	64.10	37.03	34.32	64.18	53.86	72.28	181.73	232.97
**%**	39.74	50.26	47.43	28.75	36.96	46.48	38.67	33.27	45.19	23.49	32.56	37.74	37.38	38.22	52.01	28.84	40.17	59.20	48.30
**(B) Alcohols**																			
1-Octen-3-ol	0.68	0.66	0.56	0.91	0.90	0.47	1.04	0.20	0.69	0.75	1.65	0.88	0.40	0.63	0.33	0.53	0.48	1.43	2.47
**SubTOTAL**	0.68	0.66	0.56	0.91	0.90	0.47	1.04	0.20	0.69	0.75	1.65	0.88	0.40	0.63	0.33	0.53	0.48	1.43	2.47
**%**	0.69	0.27	0.30	0.63	1.09	0.42	0.39	0.23	0.53	0.40	1.54	0.52	0.41	0.71	0.27	0.29	0.26	0.47	0.51
**(C) Esters**																			
Ethyl butyrate	-	-	-	-	-	-	5.53	-	-	21.99	5.68	8.23	4.71	-	-	1.24	-	-	-
Ethyl 2-methylbutanoate	1.52	-	-	-	-	-	-	-	-	23.17	7.90	-	3.74	-	-	-	-	-	-
Ethyl pentanoate	-	-	-	-	-	-	-	-	-	1.28	0.07	0.11	-	-	-	-	-	-	-
Ethyl hexanoate	4.68	3.13	4.48	-	1.24	2.06	12.71	-	2.06	29.47	11.32	10.75	6.08	1.72	2.57	5.81	-	-	-
**SubTOTAL**	6.19	3.13	4.48	0.00	1.24	2.06	18.24	0.00	2.06	75.90	24.97	19.08	14.53	1.72	2.57	7.05	0.00	0.00	0.00
**%**	6.35	1.27	2.41	0.00	1.51	1.84	6.88	0.00	1.58	40.44	23.40	11.23	14.67	1.91	2.09	3.78	0.00	0.00	0.00

**(E) Aldehydes**																			
3-Methylbutanal	2.32	0.48	-	-	-	-	-	2.84	-	1.46	-	1.43	1.99	-	-	1.44	-	-	-
Octanal	0.37	0.98	0.76	0.62	0.27	0.46	2.06	0.31	0.41	0.82	0.25	1.11	0.28	0.32	0.42	-	0.19	0.46	0.51
Nonanal	3.91	3.84	1.60	2.40	2.40	2.60	7.54	2.09	3.03	4.10	2.26	5.62	1.44	1.69	3.75	2.76	2.18	6.30	9.74
Decanal	-	-	-	-	-	-	1.28	-	-	-	-	-	0.40	0.57	0.67	0.63	-	-	2.41
(*E*)-2-Nonenal	-	-	-	-	-	-	-	-	0.95	-	-	2.57	-	-	2.53	1.79	-	3.35	37.44
(*E,Z*)-2,6-Nonadienal	-	-	-	-	-	5.97	5.43	-	-	-	-	-	-	-	-	-	-	-	-
**SubTOTAL**	6.61	5.30	2.37	3.01	2.67	9.04	16.30	5.23	4.39	6.38	2.51	10.75	4.11	2.58	7.38	6.62	2.37	10.11	50.10
**%**	6.77	2.15	1.27	2.07	3.24	8.06	6.14	5.91	3.37	3.40	2.35	6.33	4.15	2.87	5.98	3.54	1.32	3.29	10.39
**(F) Terpenes**																			
D-Limonene	-	1.55	2.39	0.21	0.55	1.74	11.56	6.11	2.31	4.30	2.33	2.53	1.76	2.44	0.89	1.34	0.22	-	0.30
Rose oxide I (trans)	-	-	0.45	0.27	-	-	1.84	-	-	0.18	-	0.26	-	-	-	0.33	0.37	-	1.04
Linalool	0.07	-	2.23	6.07	0.07	-	4.09	7.70	1.32	0.04	0.10	0.24	0.02	-	0.04	24.77	5.30	0.38	15.04
Geranial	0.02	0.03	0.35	2.28	0.08	-	0.13	0.07	0.04	0.02	0.10	0.41	<0.01	0.03	-	0.22	0.66	0.34	-
Citronellol	0.06	-	1.87	0.28	-	-	6.41	0.02	0.02	0.12	0.04	0.31	0.01	-	-	0.79	0.37	0.12	1.70
Nerol	0.01	-	0.46	0.26	<0.01	-	0.11	0.03	<0.01	<0.01	0.04	0.04	<0.01	-	-	0.50	1.03	0.01	0.72
Geraniol	0.23	-	4.07	2.18	0.07	-	0.85	0.77	0.18	0.06	0.24	0.30	0.07	<0.01	-	5.84	8.80	0.37	4.29
Cedrol	3.71	6.88	3.77	2.35	1.42	2.22	5.95	3.20	5.03	2.64	1.42	1.89	1.32	1.28	2.21	1.97	-	0.32	-
Geranic acid	1.43	0.32	5.25	39.96	1.65	0.72	9.50	0.92	1.86	0.69	0.21	1.65	0.30	0.14	-	29.70	52.92	5.63	38.58
**SubTOTAL**	5.53	8.78	20.84	53.86	3.84	4.68	40.46	18.83	10.76	8.06	4.48	7.64	3.49	3.89	3.14	65.46	69.66	7.17	61.67
**%**	5.67	3.56	11.23	36.91	4.65	4.18	15.25	21.26	8.26	4.29	4.20	4.50	3.52	4.33	2.54	35.05	38.71	2.34	12.78
**(G) C_{13}-Norisoprenoids**																			
β-Damascenone	7.36	28.33	22.59	21.48	12.44	9.23	23.90	10.58	12.67	10.99	16.10	15.04	8.56	15.64	7.63	23.28	15.12	47.66	68.31
β-Ionone	32.44	76.43	46.77	24.71	30.88	34.49	62.77	24.25	40.85	41.52	22.25	52.36	30.94	31.02	38.18	29.98	20.02	58.90	66.85
**SubTOTAL**	39.80	104.76	69.36	46.19	43.32	43.72	86.67	34.83	53.52	52.52	38.35	67.40	39.50	46.66	45.81	53.26	35.15	106.55	135.16
**%**	40.78	42.49	37.35	31.65	52.55	39.02	32.67	39.33	41.07	27.98	35.95	39.68	39.87	51.96	37.12	28.51	19.53	34.71	28.02
**TOTAL**	97.60	246.54	185.68	145.94	82.44	112.06	265.31	88.55	130.31	187.70	106.70	169.85	99.07	89.79	123.41	186.78	179.93	307.00	482.37

Table S5. Each class of volatile compounds as measured in the pulp juice, skin and whole grape berries in popular and unfamiliar cultivars table grapes. The results are shown as the mean values of all unfamiliar (19) and popular (20) table grapes cultivars, respectively. The popular cultivars minus unfamiliar cultivars is difference.

Compounds	Pulp juice				Skin				Whole grape berries			
	Popular Cultivars	Unfamiliar Cultivars	Difference	Percentage	Popular Cultivars	Unfamiliar cultivars	Difference	Percentage	Popular Cultivars	Unfamiliar Cultivars	Difference	Percentage
(A)C_6 compounds	622.59	585.98	36.61	6.25	775.86	887.98	-112.12	-12.63	1398.45	1473.96	-75.51	-5.12
(B)Alcohols	6.78	3.99	2.79	69.92	9.20	6.24	2.96	47.36	15.98	10.23	5.75	56.16
(C)Esters	1253.63	1220.94	32.69	2.68	415.96	403.06	12.90	3.20	1669.59	1624.00	45.59	2.81
(D)Acids	28.55	26.14	2.40	9.19	35.75	29.45	6.31	21.41	58.94	55.59	3.34	6.02
(E)Aldehydes	15.93	12.82	3.12	24.32	24.47	31.92	-7.45	-23.35	40.40	44.74	-4.34	-9.69
(F)Terpenes	125.27	79.06	46.20	58.44	2102.62	619.90	1482.72	239.19	2227.88	698.96	1528.92	218.74
(G)C_{13}-Norisoprenoids	0.20	0.24	-0.03	-14.08	0.53	0.51	0.02	3.82	0.73	0.75	-0.01	-1.84
(H)Ketones	-	0.24	-0.24	-100.00	-	0.85	-0.85	-100.00	-	1.08	-1.08	-100.00
TOTAL	2052.95	1929.41	123.54	6.40	3359.03	1979.91	1379.12	69.66	5411.98	3909.32	1502.66	38.44

Table S6. Terpenes volatile compounds as measured in skin for unfamiliar and popular cultivars table grapes. The results are shown as the mean values of all unfamiliar (19) and popular (20) table grapes cultivars, respectively. The popular cultivars minus unfamiliar cultivars is difference.

**Compounds**	**Popular Cultivars**	**Unfamiliar Cultivars**	**Difference**	**Percentage**
α-Pinene	0.08	0.05	0.03	55.45
β-Pinene	1.48	0.20	1.27	622.29
α-Phellandrene	0.80	0.50	0.30	60.11
β-Myrcene	3.83	1.75	2.08	118.77
α-Terpinene	-	0.65	-0.65	-100.00
D-Limonene	20.55	22.38	-1.83	-8.18
β-trans- Ocimene	-	0.26	-0.26	-100.00
γ-Terpinene	1.32	1.06	0.27	25.26
β-cis-Ocimene	-	0.06	-0.06	-100.00
P-Cymene	0.53	0.39	0.14	35.60
Terpinolene	3.41	2.52	0.88	34.87
Rose oxide II (cis)	0.07	0.00	0.07	-
Rose oxide I (trans)	0.14	0.12	0.02	13.84
cis-Linalool oxide	-	0.01	-0.01	-100.00
Nerol oxide	0.92	0.25	0.66	263.34
Citronellal	-	0.01	-0.01	-100.00
Linalool	54.11	21.31	32.80	153.91
4-Terpineol	0.86	1.42	-0.57	-39.88
Hotrienol	0.71	0.17	0.54	312.25
Menthol	-	0.15	-0.15	-100.00
Neral	10.69	4.72	5.97	126.63
α-Terpineol	23.13	12.23	10.90	89.13
Geranial	17.43	8.07	9.36	116.01
Citronellol	28.04	25.53	2.51	9.83
Myrtenol	0.03	0.05	-0.03	-48.62
Nerol	137.92	51.23	86.69	169.21
Geraniol	362.45	59.61	302.84	508.02
E-Nerolidol	0.06	0.01	0.05	475.00
Cedrol	2.35	1.25	1.10	87.73
Eugenol	-	0.20	-0.20	-100.00
Geranic acid	1431.71	403.00	1028.70	255.26
**TOTAL**	2102.62	619.64	1482.98	239.33

Table S7. Chemical standards, retention index (RI), odour descriptors, odorant series, odour threshold (μg/l) of the studied compounds. Notes: LRI, linear retention index on a HP-INNOWAX column. The odour threshold and odour descriptors were reported in literature. Compounds determined in water solution, except for 2-ethyl hexanol, propyl acetate, hexyl acetate, ethyl 3-hydroxybutyrate determined in ethanol-water solution; (*Z*)-3-hexenyl acetate determined in sunflower oil; geranic acid was the same as geraniol; hotrienol not found the media. Primary series: 1, herbaceous; 2, floral; 3, fruity; 4, sweet; 5, spicy; 6, roasty; 7, fatty; 8, earthy; 9, balsamic; 10, solvent. Secondary series: 2-1, flower; 2-2, rose; 2-3, camomile; 2-4, fragant; 2-5, lavender; 2-6, geranium; 2-7, hyacinth; 2-8, lilac; 2-9, lily; 2-10, violet; 2-11, orange flower; 2-12, geranium; 2-13, magnolia; 3-1, fruity; 3-2, apple; 3-3, cherry; 3-4, pineapple; 3-5, banana; 3-6, strawberry; 3-7, pear; 3-8, apricot; 3-9, grape; 3-10, coconut; 3-11, citrus; 3-12, lemon; 3-13, orange; 3-14, raspberry; 4-1, sweet; 4-2, honey; 4-3, caramel; 4-4, burnt sugar; 4-5, marshmallow.

**Compounds**	**Odour Threshold (μg/l)**	**Odour Descriptor**	**Primary Series**	**Secondary Series**
**C_6 compounds**				
Hexanal	4.5[1]	Green[2]	1	
(*Z*)-3-Hexenal	0.25[3]	Grass[4]	1	
(*E*)-2-Hexenal	17[5]	Grass[5], herbaceous[5]	1	
Hexanol	500[1, 6]	Flower[5, 7-8], green[5, 7-8], cut grass[7-8], grass[5] , herbaceous[5, 9-10], wood[5, 9-10]	1,2	2-1
(*E*)-3-Hexenol	1000[11]	Green[10, 12], bitter[12], fatty[12], herbaceous[10], fresh[11]	1,7	
(*Z*)-3-Hexenol	70[1]	Grass[4-5, 13], herbaceous[5, 10], green[5, 7-8, 14-15], fatty[5, 12, 14], bitter[5, 12, 14]	1,7	
(*E*)-2-Hexenol	100[16]	Herbaceous[5, 10], green[5, 10, 16]	1	
**Alcohols**				
2-Methyl-3-butene-2-ol	10[17]	Fruity[17], faint scent[17]	3,7	3-1
3-Methyl-2-butanol	1259.9[18]	Green apple[18], solvent[18]	3,10	3-2
Pentanol	4000[1]	Fatty[8]	7	
Butanol	500[1]	Medicinal[8, 14-15], phenolic[8]	7,9,10	
2-Heptanol	70[16]	Fruity[16], herbaceous[16]	1,3	3-1
1-Octen-3-ol	1[17]	Mushroom[2, 17]	8	
Heptanol	425[1]	Oily[8]	7	
2-Ethyl hexanol	270[19]	Floral[20]	2	
Octanol	110[21]	Jasmine[9], lemon[9]	2	
Nonanol	50[21]	Rose-orange[16]	2	
Benzyl alcohol	10000[22]	Roasted[5], toasted[5], sweet[5, 8], fruity[5, 8], cherry[23]	3,4,6	3-3, 4-1
Phenylethyl alcohol	1100[22]	Floral[5, 8], rose[5, 8], honey[5]	2,4	2-2, 4-2
**Esters**				
Ethyl acetate	5000[1]	Pineapple[10, 24], fruity[8, 10], solvent[8, 10], anise[24], balsamic[9]	3,5,7,9,10	3-4
Ethyl propionate	10[1]	Banana[9], apple[9], strawberry[25]	3	3-2, 3-5, 3-6
Ethyl isobutyrate	0.1[1]	Fruity[9], strawberry[4]	3	3-6
Propyl acetate	4700[14]	Celery[14]	1	
Ethyl butyrate	1[1]	Fruity[8], strawberry[13], apple[26], banana[26], pineapple[26]	3	3-2, 3-4, 3-5, 3-6
Ethyl 2-methylbutanoate	0.091[16]	Banana[27], apple[27], pineapple[28], strawberry[28]	3	3-2, 3-4, 3-5, 3-6
Ethyl 3-methylbutanoate	0.1[1]	Fruity[2], apple[29]	3	3-2
Butyl acetate	66[1]	Fruity[14], apple[30], pear[31], pineapple[23]	3	3-2, 3-5, 3-7
Ethyl pentanoate	1.5[3]	Grass[4]	1	
(*Z*)-2-Butenoic acid ethyl ester	13.6[32]	Fruity[32], cooked apple[33]	3	3-2
Methyl hexanoate	70[34]	Fruity[27], apricot[27], pineapple[27], sweet[34]	3,4	3-4, 3-8, 4-1
Ethyl hexanoate	1[1]	Fruity[9, 14], green apple[7-9, 14], banana[9, 14], wine-like[9, 14], brandy[14]	3	3-2, 3-5

Hexyl acetate	670[10, 14]	Apple[10, 14], pear[10, 13-14], floral[7-8, 10, 14], green[7-8], cherry[13-14]	1,2,3	2-1, 3-2, 3-3, 3-7
(*Z*)-3-Hexenyl acetate	750[35]	Fruity[35], green leaves[35], banana[36]	1,3	3-5
Ethyl heptanoate	2[1]	Winelike[12], brandy[12], fruity[12], banana[37], strawberry[37]	3,10	3-5, 3-6
Ethyl octanoate	194[1]	Sweet[8-9], floral[9], fruity[8-9], banana[9], pear[9], brandy[9], pineapple[12],	2,3,4	2-1, 3-4, 3-5, 3-7, 4-1
Ethyl 3-hydroxybutyrate	20000[13]	Grape[12], fruity[15], caramel[12], toasted[12]	3,4,6	3-9
Benzoic acid ethyl ester	60[1]	Floral[38-39], camomile[38], fruity[39]		2-3, 3-1
Methyl salicylate	40[1]	Green[16]	1	
**Acids**				
Hexanoic acid	3000[40]	Rancid[9], cheese[9], fatty[9], Sweat[8]	7	
2-Hexenoic acid	1000[16]	Fatty[16], rancid[16, 41]	7	
Octanoic acid	3000[1]	Rancid[9], cheese[8-9], fatty[9], sweat[8]	7	
Nonanoic acid	3000[1]	Coconut[26], fatty[26]	3,7	3-10
Decanoic acid	10000[1]	Fatty[9], rancid[9]	7	
**Aldehydes**				
2-Methylbutanal	1.3[16]	Green[16], malty[16]	1	
3-Methylbutanal	0.2[40]	Fresh grass[16], cocoa[16]	1	
Pentanal	12[1, 21]	Fat[42], green[42]	1,7	
Heptanal	3[30]	Fat[30], citrus[30], rancid[30]	3,7	3-11
Octanal	0.7[1, 3, 21]	Honey[12], green[12, 41], fatty[12], fruity[2], citrus[2], lemon[4, 41], fat[41], soap[41], flower[43]	1,2,3,4,7	3-11, 3-12, 4-2, 2-1
Nonanal	1[1, 21]	Fat[41], citrus[41], green[41], fruity[2], orange peel[44]	1,3	3-11, 3-13
(*E*)-2-Octenal	3[1, 6, 21]	Green[41], nut[41], fat[41]	1	
Benzaldehyde	350[1]	Sweet[7-8], fruity[7-8], cherry[45], roasted[10], almond[10, 13-14], fragant[14], burnt sugar[13]	2,3,4,6	2-4, 3-3, 4-4
(*E*)-2-Nonenal	0.08[1]	Wet[4], earth[4], fatty[46], hay-like[46]	7,8	
(*E*,*Z*)-2,6-Nonadienal	0.02[1]	Green[2], fatty[47], vegetative[47]	1,7	
Phenylacetaldehyde	4[1]	Flowery[4], rose[4], honey[30], sweet[30]	2,4	2-2, 4-2
**Terpenes**				
α-Pinene	6[1]	Pine[16], resinous[16]	1	
β-Pinene	140[1]	Woody[16], resinous[16]	1	
α-Phellandrene	40[1]	Sweet[16], rose-like[16]	2,4	2-2, 4-1
β-Myrcene	36[22]	Green burning[2], green[2]	1,6	
α-Terpinene	85[48]	Herbaceous[39]	1	
D-Limonene	10[22]	Fruity[49], lemon[30, 49], orange[30], citrus-like[50]	3	3-11, 3-12, 3-13
Eucalyptol	12[17]	Camphoric[17]	1	
β-Phellandrene	36[51]	Herbaceous[52], turpentine[52], terpenic[52], minty[52]	1	
trans-β-Ocimene	34[52]	Green[53], terpenic[53], mild[53], citrus[53], sweet[53], orange[53], lemon[53]	1,3,4	3-11, 3-12, 3-13, 4-1
γ-Terpinene	1000[1]	Fruity[16], lemon-like[16], citrus[54]	3	3-11, 3-12
β-cis-Ocimene	34[48]	Herbaceous[55], citrus-like[55], citrus[2], minty[2]	1,3	3-11
P-Cymene	11.4[1]	Citrus[2], green[2], lemon[36], fruity[36]	1,3	3-11, 3-12
Terpinolene	200[1]	Piney[56]	1	
Rose oxide II (cis)	0.5[22]	Floral[2], lychee-like[2], rose[57]	2	2-2
Rose oxide I (trans)	0.5[22]	Rose[57]	2	2-2
cis-Linalool oxide	320[1]	Floral[2], green[2], flower[58], lavender[58]	1,2	2-5
Nerol oxide	3000[59]	Oil[59], flower[59], geranium[59]	2,7	2-6
Citronellal	46[60]	Solvent[2], lemon[2, 60], green[60], grass[60]	1,3,10	3-12
Linalool	6[22]	Citrus[9, 14-15], floral[8-9, 14-15], sweet[9, 14-15], grape-like[9, 14-15], marshmallow[61], fruity[61], rose[61], flower[36], lavender[36]	2,3,4	2-2, 2-5, 3-9, 3-11, 4-5
4-Terpineol	130[1]	Flowers[11, 49], nutmeg[11], moldy[10]	1,2,5	2-1
Hotrienol	110[62]	Fresh[45, 62], floral[62], fruity[62], hyacinth[36, 45], lemon[45]	1,2,3	2-7, 3-12
Menthol	920[48]	Minty[2]	1	
Neral	1000[1]	Fruity[9], lemon[30], citrus-like[25]	3	3-11, 3-12
α-Terpineol	330[22]	Lilac[14-15], floral[14-15], sweet[10, 14-15], lily[10], cake[10]	2,4	2-8, 2-9, 4-1
Geranial	32[1]	Citrus[2], citric fruit[2], lemon[30], mint[30]	3	3-11, 3-12
Citronellol	40[22]	Rose[9, 13]	2	2-2
Myrtenol	7[16]	Flowery[16], mint[16]	1,2	2-1
Nerol	300[22]	Flower[13], grass[13], floral[12, 63], green[12], violets[63], rose[5]	1,2	2-2, 2-10

Geraniol	40[22]	Citric[5], floral[5, 15], orange flower[5, 15], roses[5, 7-8, 13], geranium[5, 7-8, 13]	2	2-2, 2-11, 2-12
E-Nerolidol	250[1]	Rose[12, 14], apple[12, 14], green[12, 14], citrus[14], waxy[12], woody[12]	1,2,3,10	2-2, 3-2, 3-11
Cedrol	0.5[17]	Cool[17], camphor[41]	1	
Eugenol	6[1]	Spices[8], clove[8], honey[8]	4,5	4-2
Geranic acid	40 [64]	Green[64]	1	
**C_{13}-Norisoprenoids**				
β-Damascenone	0.002[40]	Sweet[2, 7-8], fruity[7-8], floral[2], honey[2, 27], baked apple[4], apple[27], rose[27]	2,3,4	3-2, 2-2, 2-6, 4-2
Geranyl acetone	60[65]	Fresh[65], floral[65], magnolia[54], green[54]	1,2	2-13
trans-β-Ionone	0.007[1, 40]	Balsamic[14], rose[14], violet[14]	2,9	2-2, 2-10
**Ketones**				
2-Octanone	50[21]	Bitter[66], green[66], fat[45], fragant[45], gasoline[45], mold[45], soap[45]	1,7	
6-Methyl-5-hepten-2-one	50[1]	Fruity[67], orange[67], citrus[45], mushroom[45], pepper[45], rubber[45], strawberry[45], green[47], lemony[47], raspberry[68]	1,3,8	3-6, 3-11, 3-12, 3-13, 3-14

Table S8. Significant differences of predicted aroma consumer liking scores by ANOVA analysis followed by Duncan's test (*p* = 0.05). Note: Cultivars in different columns had significant difference. Capital and small letters refer to the table grape cultivars as listed in **Figure 4**.

Cultivars	Subsets (alpha = 0.05)																		
	**1**	**2**	**3**	**4**	**5**	**6**	**7**	**8**	**9**	**10**	**11**	**12**	**13**	**14**	**15**	**16**	**17**	**18**	**19**
b	1.97																		
i		2.31																	
A			2.80																
N				3.52															
l				3.53															
g				3.55	3.55														
Q				3.63	3.63	3.63													
R				3.65	3.65	3.65	3.65												
K				3.72	3.72	3.72	3.72	3.72											
F				3.73	3.73	3.73	3.73	3.73	3.73										
M				3.75	3.75	3.75	3.75	3.75	3.75	3.75									
H				3.81	3.81	3.81	3.81	3.81	3.81	3.81	3.81								
p					3.83	3.83	3.83	3.83	3.83	3.83	3.83								
o						3.89	3.89	3.89	3.89	3.89	3.89	3.89							
h						3.92	3.92	3.92	3.92	3.92	3.92	3.92	3.92						
n						3.92	3.92	3.92	3.92	3.92	3.92	3.92	3.92						
k							3.94	3.94	3.94	3.94	3.94	3.94	3.94						
m							3.95	3.95	3.95	3.95	3.95	3.95	3.95						
q							3.95	3.95	3.95	3.95	3.95	3.95	3.95						
r								3.97	3.97	3.97	3.97	3.97	3.97						
C								4.00	4.00	4.00	4.00	4.00	4.00						
O								4.00	4.00	4.00	4.00	4.00	4.00						
D									4.03	4.03	4.03	4.03	4.03						
B										4.05	4.05	4.05	4.05	4.05					
E											4.09	4.09	4.09	4.09	4.09				
L												4.14	4.14	4.14	4.14	4.14			
J												4.16	4.16	4.16	4.16	4.16			
c													4.19	4.19	4.19	4.19	4.19		
d													4.19	4.19	4.19	4.19	4.19		
t													4.20	4.20	4.20	4.20	4.20		
f														4.33	4.33	4.33	4.33		
s														4.34	4.34	4.34	4.34		
j															4.36	4.36	4.36		
S																4.39	4.39		
I																4.43	4.43		
a																	4.47		
e																		4.83	
G																		5.08	
P																			5.44

Table S9. The key aroma compounds correlated with predicted aroma consumer liking selected by variable selection procedure (VIP > 1) using OPLS regression model, their VIP scores and coefficients.

Key Compounds	VIPpred	Coefficient
β-Ionone	1.9824	0.0945
Nonanal	1.9336	0.2335
Linalool	1.7686	0.0189
Cedrol	1.7362	0.0692
Terpinolene	1.6259	−0.0046
Octanal	1.5987	−0.0006
Geranic acid	1.5933	0.1483
α-Terpineol	1.5728	−0.0324
P-Cymene	1.5322	0.1371
2-Hexenoic acid	1.5304	0.2182
(*E*)-2-Octenal	1.5040	0.0738
(*E*)-2-Hexenal	1.4670	0.0276
Neral	1.4330	0.0464
Nerol	1.4236	−0.0498
Geranial	1.4010	0.0617
Hexanal	1.3661	0.0801
3-Methylbutanal	1.3383	−0.0002
Geraniol	1.3316	−0.0448
α-Phellandrene	1.2941	0.0494
2-Methylbutanal	1.2105	0.0792
γ-Terpinene	1.1947	0.0381
Hotrienol	1.1938	−0.0122
E-Nerolidol	1.1607	0.1413
Rose oxide I (trans)	1.1326	0.0129
Nerol oxide	1.1049	−0.0511
β-Pinene	1.0097	−0.0193

Table S10. The results of OPLS models established by primary aromatic series values.

Attribute	No. of Latent Variable	R^2X	R^2Y	Q^2Y	R^2	RMSEE	RMSEcv
Aromatic series	1 + 0 + 0	0.566	0.499	0.438	0.4989	0.4741	0.4938

Table S11. The average concentrations and percentage of different types compounds in 39 table grapes.

Compounds	Concentrations (Mg/kg)	Percentage （%）
(A) C_6 alcohols	1435.24	30.67
(B) Alcohols	13.18	0.28
(C) Esters	1647.38	35.20
(D) Acids	57.31	1.22
(E)Aldehydes	42.52	0.91
(F) Terpenes	1483.03	31.69
(G) C_{13}-Norisoprenoids	0.74	0.02
(H) Ketones	0.53	0.01
TOTAL	4679.91	

Table S12. Information for unfamiliar table grape cultivars in this study.

Unfamiliar Cultivars	Codes	Varieties	Parents
Yongyou 1 #	A	Hybrids between V. viniferaand V. labrusca	Bud mutation of Kyoho
Ryogyoku	B	Hybrids between V. viniferaand V. labrusca	S9110×Neo-Muscat
Summer Black	C	Hybrids between V. viniferaand V. labrusca	Kyoho × Thompson Seedless
Muscat Duke Amour	D	V. vinifera	Unknown
Cannon Hall Muscat	E	V. vinifera	4 Time of body bud mutation of Muscat of Alexandria
Baby Finger	F	V. vinifera	Black Swan×Pizzutei
Xiangyue	G	Hybrids between V. viniferaand V. labrusca	7601（Bud mutation of Muscat Hamburg）×8001(Bud mutation of Zixiangshui)
Jintianmeigui	H	V. vinifera	Muscat Hamburg×Red Globe
Katta Kourgan	I	V. vinifera	Unknown
Aki Queen	J	Hybrids between V. viniferaand V. labrusca	Kyoho Seedling
Honney Black	K	Hybrids between V. viniferaand V. labrusca	Kyoho Seedling
Ougyoku	L	Hybrids between V. viniferaand V. labrusca	Moli Seedling
Benifuji	M	Hybrids between V. viniferaand V. labrusca	Gold Muscat×Kuroshio
Rosario Rosso	N	V. vinifera	Rosario Bianco×Buby Okuyama
Lilit	O	Unknown	Introduction from Israel
Heikuixiang	P	Hybrids between V. viniferaand V. labrusca	Kyoho×Shengyang Muscat
Princess Seedles	Q	Unknown	Unknown
Shinano Smile	R	Hybrids between V. viniferaand V. labrusca	Takasumi Seedling
Royal	S	V. vinifera	Mutation of Alphonse Lavallee

Supplemental References list

1. Pino, J. A.; Mesa, J., Contribution of volatile compounds to mango (Mangifera indica L.) aroma. *Flavour and Fragrance J.* **2006,** *21*, 207-213.
2. Mahattanatawee, K.; Perez-Cacho, P. R.; Davenport, T.; Rouseff, R., Comparison of three lychee cultivar odor profiles using gas chromatography-olfactometry and gas chromatography-sulfur detection. *Journal of agricultural and Food Chem.* **2007,** *55*, 1939-1944.
3. Genovese, A.; Dimaggio, R.; Lisanti, M. T.; Piombino, P.; Moio, L., Aroma composition of red wines by different extraction methods and Gas Chromatography-SIM/MASS spectrometry analysis. *Ann Chim* **2005,** *95* (6), 383-94.
4. Cullere, L.; Escudero, A.; Cacho, J.; Ferreira, V., Gas chromatography-olfactometry and chemical quantitative study of the aroma of six premium quality spanish aged red wines. *Journal of agricultural and food chemistry* **2004,** *52* (6), 1653-60.
5. Genovese, A.; Lamorte, S. A.; Gambuti, A.; Moio, L., Aroma of Aglianico and Uva di Troia grapes by aromatic series. *Food Research Int.* **2013,** *53* (1), 15-23.
6. Buttery, R.; Seifert, R.; Guadagni, D.; Ling, L., Characterization of additional volatile components of tomato. *Journal of agricultural and food chemistry* **1971,** *19* (3), 524-529.
7. Garcia-Carpintero, E. G.; Sanchez-Palomo, E.; Gomez Gallego, M. A.; Gonzalez-Vinas, M. A., Effect of cofermentation of grape varieties on aroma profiles of la mancha red wines. *J Food Sci* **2011,** *76* (8), C1169-80.
8. García-Carpintero, E. G.; Sánchez-Palomo, E.; Gallego, M. A. G.; González-Viñas, M. A., Volatile and sensory characterization of red wines from cv. Moravia Agria minority grape variety cultivated in La Mancha region over five consecutive vintages. *Food Research Int.* **2011,** *44* (5), 1549-1560.
9. Peinado, R. A.; Mauricio, J. C.; Moreno, J., Aromatic series in sherry wines with gluconic acid subjected to different biological aging conditions by Saccharomyces cerevisiae var. capensis. *Food Chemistry* **2006,** *94* (2), 232-239.
10. Franco, M.; Peinado, R. A.; Medina, M.; Moreno, J., Off-vine grape drying effect on volatile compounds and aromatic series in must from Pedro Ximénez grape variety. *Journal of agricultural and food chemistry* **2004,** *52* (12), 3905-3910.
11. Noguerol-Pato, R.; Gonzalez-Alvarez, M.; Gonzalez-Barreiro, C.; Cancho-Grande, B.; Simal-Gandara, J., Evolution of the aromatic profile in Garnacha Tintorera grapes during raisining and comparison with that of the naturally sweet wine obtained. *Food Chemistry* **2013,** *139* (1-4), 1052-1061.
12. Moyano, L.; Zea, L.; Moreno, J.; Medina, M., Analytical study of aromatic series in sherry wines subjected to biological aging. *Journal of agricultural and food chemistry* **2002,** *50* (25), 7356-61.
13. González Álvarez, M.; González-Barreiro, C.; Cancho-Grande, B.; Simal-Gándara, J., Relationships between< i> Godello</i> white wine sensory properties and its aromatic fingerprinting obtained by GC–MS. *Food Chemistry* **2011,** *129* (3), 890-898.
14. Peinado, R. A.; Moreno, J.; Bueno, J. E.; Moreno, J. A.; Mauricio, J. C., Comparative study of aromatic compounds in two young white wines subjected to pre-fermentative cryomaceration. *Food Chemistry* **2004,** *84* (4), 585-590.
15. Gómez-Míguez, M. J.; Gómez-Míguez, M.; Vicario, I. M.; Heredia, F. J., Assessment of colour and aroma in white wines vinifications: Effects of grape maturity and soil type. *Journal of Food Engineering* **2007,** *79* (3), 758-764.
16. Qian, M. C.; Wang, Y., Seasonal variation of volatile composition and odor activity value of 'Marion'(Rubus spp. hyb) and 'Thornless Evergreen'(R. laciniatus L.) blackberries. *Journal of food science* **2005,** *70* (1), C13-C20.
17. Yang, C.; Luo, L.; Zhang, H.; Yang, X.; Lv, Y.; Song, H., Common aroma-active components of propolis from 23 regions of China. *J Sci Food Agric* **2010,** *90* (7), 1268-82.
18. Giri, A.; Osako, K.; Okamoto, A.; Ohshima, T., Olfactometric characterization of aroma active compounds in fermented fish paste in comparison with fish sauce, fermented soy paste and sauce products. *Food Research Int.* **2010,** *43* (4), 1027-1040.
19. Pino, J. A.; Queris, O., Analysis of volatile compounds of mango wine. *Food Chemistry* **2011,** *125* (4), 1141-1146.
20. Fan, W.; Xu, Y.; Jiang, W.; Li, J., Identification and quantification of impact aroma compounds in 4 nonfloral Vitis vinifera varieties grapes. *J Food Sci* **2010,** *75* (1), S81-8.
21. Buttery, R. G.; Turnbaugh, J. G.; Ling, L. C., Contribution of volatiles to rice aroma. *Journal of agricultural and food chemistry* **1988,** *36* (5), 1006-1009.
22. Fenoll, J.; Manso, A.; Hellin, P.; Ruiz, L.; Flores, P., Changes in the aromatic composition of the Vitis vinifera grape Muscat Hamburg during ripening. *Food Chemistry* **2009,** *114* (2), 420-428.
23. Qian, M. C.; Wang, Y., Seasonal Variation of Volatile Composition and Odor Activity Value of'Marion'(Rubus

spp. hyb) and'Thornless Evergreen'(R. laciniatus L.) Blackberries. *Journal of Food Science* **2005,** *70* (1), C13–C20.
24. Ruiz, M. J.; Zea, L.; Moyano, L.; Medina, M., Aroma active compounds during the drying of grapes cv. Pedro Ximenez destined to the production of sweet Sherry wine. *European Food Research and Technology* **2010,** *230* (3), 429-435.
25. Cuevas, F. J.; Moreno-Rojas, J. M.; Ruiz-Moreno, M. J., Assessing a traceability technique in fresh oranges (Citrus sinensis L. Osbeck) with an HS-SPME-GC-MS method. Towards a volatile characterisation of organic oranges. *Food Chem* **2017,** *221*, 1930-1938.
26. Song, C.-Z.; Liu, M.-Y.; Meng, J.-F.; Shi, P.-B.; Zhang, Z.-W.; Xi, Z.-M., Influence of foliage-sprayed zinc sulfate on grape quality and wine aroma characteristics of Merlot. *European Food Research and Technology* **2015,** *242* (4), 609-623.
27. Bellincontro, A.; Matarese, F.; D'Onofrio, C.; Accordini, D.; Tosi, E.; Mencarelli, F., Management of postharvest grape withering to optimise the aroma of the final wine: A case study on Amarone. *Food Chem* **2016,** *213*, 378-87.
28. Corral, S.; Salvador, A.; Flores, M., Elucidation of key aroma compounds in traditional dry fermented sausages using different extraction techniques. *J Sci Food Agric* **2015,** *95* (6), 1350-61.
29. Vilanova, M.; Genisheva, Z.; Bescansa, L.; Masa, A.; Oliveira, J. M., Volatile composition of wines from cvs. Blanco lexítimo, Agudelo and Serradelo (Vitis vinifera) grown in Betanzos (NW Spain). *Journal of the Institute of Brewing* **2009,** *115* (1), 35-40.
30. Wang, L.; Baldwin, E. A.; Plotto, A.; Luo, W.; Raithore, S.; Yu, Z.; Bai, J., Effect of methyl salicylate and methyl jasmonate pre-treatment on the volatile profile in tomato fruit subjected to chilling temperature. *Postharvest Biology and Technology* **2015,** *108*, 28-38.
31. Du, X. F.; Kurnianta, A.; McDaniel, M.; Finn, C. E.; Qian, M. C., Flavour profiling of 'Marion' and thornless blackberries by instrumental and sensory analysis. *Food Chemistry* **2010,** *121* (4), 1080-1088.
32. Lasekan, O.; Khatib, A.; Juhari, H.; Patiram, P.; Lasekan, S., Headspace solid-phase microextraction gas chromatography–mass spectrometry determination of volatile compounds in different varieties of African star apple fruit (Chrysophillum albidum). *Food Chemistry* **2013,** *141* (3), 2089-2097.
33. Silva, G. D.; Hj, C. D. N., Complementary use of hyphenated purge-and-trap gas chromatography techniques and sensory analysis in the aroma profiling of strawberries (Fragaria ananassa). *Journal of Agricultural & Food Chemistry* **1999,** *47* (11), 4568-73.
34. Forero, D. P.; Orrego, C. E.; Peterson, D. G.; Osorio, C., Chemical and sensory comparison of fresh and dried lulo (Solanum quitoense Lam.) fruit aroma. *Food Chem* **2015,** *169*, 85-91.
35. Aparicio, R.; Morales, M. T., Characterization of olive ripeness by green aroma compounds of virgin olive oil. *Journal of agricultural and food chemistry* **1998,** *46*, 1116-1122.
36. Zhang, S.; Petersen, M. A.; Liu, J.; Toldam-Andersen, T. B., Influence of Pre-Fermentation Treatments on Wine Volatile and Sensory Profile of the New Disease Tolerant Cultivar Solaris. *Molecules* **2015,** *20* (12), 21609-25.
37. Ruiz, M. J.; Moyano, L.; Zea, L., Changes in aroma profile of musts from grapes cv. Pedro Ximenez chamber-dried at controlled conditions destined to the production of sweet Sherry wine. *LWT - Food Science and Technology* **2014,** *59* (1), 560-565.
38. Rodríguez Madrera, R.; Pando Bedriñana, R.; Suárez Valles, B., Production and characterization of aroma compounds from apple pomace by solid-state fermentation with selected yeasts. *LWT - Food Science and Technology* **2015,** *64* (2), 1342-1353.
39. Kim, B. H.; Park, S. K., Volatile aroma and sensory analysis of black raspberry wines fermented by different yeast strains. *Journal of the Institute of Brewing* **2015,** *121* (1), 87–94.
40. Buttery, R. G.; Teranishi, R.; Ling, L. C.; Turnbaugh, J. G., Quantitative and sensory studies on tomato paste volatiles. *Journal of agricultural and food chemistry* **1990,** *38* (1), 336-340.
41. Chen, L.; Zhang, X.; Jin, Q.; Yang, L.; Li, J.; Chen, F., Free and Bound Volatile Chemicals in Mulberry (Morus atropurpurea Roxb.). *J Food Sci* **2015,** *80* (5), C975-82.
42. Mallia, S.; Escher, F.; Dubois, S.; Schieberle, P.; Schlichtherle-Cerny, H., Characterization and Quantification of Odor-Active Compounds in Unsaturated Fatty Acid/Conjugated Linoleic Acid (UFA/CLA)-Enriched Butter and in Conventional Butter during Storage and Induced Oxidation. *J.agricultural and Food Chem.* **2009,** *57* , 7464-7472.
43. Peinado, I.; Rosa, E.; Heredia, A.; Escriche, I.; Andrés, A., Influence of storage on the volatile profile, mechanical, optical properties and antioxidant activity of strawberry spreads made with isomaltulose. *Food Biosci.* **2016,** *14*, 10-20.
44. González-Cebrino, F.; García-Parra, J.; Ramírez, R., Aroma profile of a red plum purée processed by high hydrostatic pressure and analysed by SPME–GC/MS. *Innovative Food Sci. Emerging Technol.* **2015,** *33*, 108-114.
45. Kim, Y.; Lee, K. G.; Kim, M. K., Volatile and non-volatile compounds in green tea affected in harvesting time and their correlation to consumer preference. *J. Food Sci. Technol.* **2016,** *53*, 3735-3743.

46. Kiatbenjakul, P.; Intarapichet, K. O.; Cadwallader, K. R., Characterization of potent odorants in male giant water bug (Lethocerus indicus Lep. and Serv.), an important edible insect of Southeast Asia. *Food Chem* **2015,** *168*, 639-647.
47. Cheong, M. W.; Liu, S. Q.; Zhou, W.; Curran, P.; Yu, B., Chemical composition and sensory profile of pomelo (Citrus grandis (L.) Osbeck) juice. *Food Chem.* **2012,** *135*, 2505-2513.
48. Xiaofen, D.; ChadE, F.; MichaelC, Q., Volatile composition and odour-activity value of thornless 'Black Diamond' and 'Marion' blackberries. *Food Chem.* **2010,** *119*, 1127-1134.
49. Noguerol-Pato, R.; Gonzalez-Alvarez, M.; Gonzalez-Barreiro, C.; Cancho-Grande, B.; Simal-Gandara, J., Aroma profile of Garnacha Tintorera-based sweet wines by chromatographic and sensorial analyses. *Food Chem.* **2012,** *134*, 2313-25.
50. San, A. T.; Joyce, D. C.; Hofman, P. J.; Macnish, A. J.; Webb, R. I.; Matovic, N. J.; Williams, C. M.; Voss, J. J. D.; Wong, S. H.; Smyth, H. E., Stable isotope dilution assay (SIDA) and HS-SPME-GCMS quantification of key aroma volatiles for fruit and sap of Australian mango cultivars. *Food Chem.*
51. Eglė Bylaitė; ‡, J. P. R.; Aagje Legger; Posthumus§, M. A., Dynamic Headspace−Gas Chromatography−Olfactometry Analysis of Different Anatomical Parts of Lovage (Levisticum officinale Koch.) at Eight Growing Stages. *J. Agricultural Food Chem.* **2000,** *48*, 6183-90.
52. Bonneau, A.; Boulanger, R.; Lebrun, M.; Maraval, I.; Gunata, Z., Aroma compounds in fresh and dried mango fruit (Mangifera indica L. cv. Kent): impact of drying on volatile composition. *I Int. J. Food Sci. Technol.* **2016,** *51*, 789-800.
53. Baranauskienė, R.; Venskutonis, P. R.; Demyttenaere, J. C. R., Sensory and instrumental evaluation of sweet marjoram (Origanum majorana L.) aroma. *Flavour and Fragrance J.***2005,** *20*, 492-500.
54. Fukuda, T.; Okazaki, K.; Shinano, T., Aroma characteristic and volatile profiling of carrot varieties and quantitative role of terpenoid compounds for carrot sensory attributes. *J. Food Sci.* **2013,** *78*, S1800-S1806.
55. Goncalves, J. L.; Figueira, J. A.; Rodrigues, F. P.; Ornelas, L. P.; Branco, R. N.; Silva, C. L.; Camara, J. S., A powerful methodological approach combining headspace solid phase microextraction, mass spectrometry and multivariate analysis for profiling the volatile metabolomic pattern of beer starting raw materials. *Food Chem* **2014,** *160*, 266-280.
56. Pino, J. A., Odour-active compounds in mango (Mangifera indica L. cv. Corazón). *Int. J. Food Sci. Technol.* **2012,** *47*, 1944-1950.
57. Wu, Y.; Zhu, B.; Tu, C.; Duan, C.; Pan, Q., Generation of volatile compounds in litchi wine during winemaking and short-term bottle storage. *J. agricultural and Food Chem.* **2011,** *59*, 4923-4931.
58. Zhu, J.; Chen, F.; Wang, L.; Niu, Y.; Chen, H.; Wang, H.; Xiao, Z., Characterization of the Key Aroma Volatile Compounds in Cranberry (Vaccinium macrocarpon Ait.) Using Gas Chromatography-Olfactometry (GC-O) and Odor Activity Value (OAV). *Journal of agricultural and food chemistry* **2016,** *64*, 4990-4999.
59. Bowen, A. J.; Reynolds, A. G., Odor potency of aroma compounds in Riesling and Vidal blanc table wines and icewines by gas chromatography-olfactometry-mass spectrometry. *Journal of agricultural and food chemistry* **2012,** *60*, 2874-83.
60. Cheong, M.; Liu, S.; Yeo, J.; Chionh, H.; Pramudya, K.; Curran, P.; Yu, B., Identification of Aroma-Active Compounds in Malaysian Pomelo ((L.) Osbeck) Peel by Gas Chromatography-Olfactometry. *J. Essential Oil Research* **2012,** *23*, 34-42.
61. Hu, K.; Zhu, X. L.; Mu, H.; Ma, Y.; Ullah, N.; Tao, Y. S., A novel extracellular glycosidase activity from Rhodotorula mucilaginosa: its application potential in wine aroma enhancement. *Letters in applied microbiology* **2016,** *62*, 169-176.
62. Castro-Vázquez, L.; Díaz-Maroto, M.; Pérez-Coello, M., Aroma composition and new chemical markers of Spanish citrus honeys. *Food Chem.* **2007,** *103*, 601-606.
63. Cai, J.; Zhu, B. Q.; Wang, Y. H.; Lu, L.; Lan, Y. B.; Reeves, M. J.; Duan, C. Q., Influence of pre-fermentation cold maceration treatment on aroma compounds of Cabernet Sauvignon wines fermented in different industrial scale fermenters. *Food Chemistry* **2014,** *154*, 217-229.
64. Noguerol-Pato, R.; González-Barreiro, C.; Cancho-Grande, B.; Martínez, M.; Santiago, J.; Simal-Gándara, J., Floral, spicy and herbaceous active odorants in Gran Negro grapes from shoulders and tips into the cluster, and comparison with Brancellao and Mouratón varieties. *Food Chem.* **2012,** *135*, 2771-2782.
65. Lasekan, O.; See, N. S., Key volatile aroma compounds of three black velvet tamarind (Dialium) fruit species. *Food Chem* **2015,** *168*, 561-5.
66. †, S. S. W.; †, S. Z.; Hedelund, P. I.; Petersen, M. A.; Byrne, D. V., Application of the fast sensory method 'Rate-All-That-Apply' in chocolate Quality Control compared with DHS-GC-MS. *Int. J. Food Sci. Technol.* **2016,** *51*, 1877-1887.
67. Bordiga, M.; Piana, G.; Coïsson, J. D.; Travaglia, F.; Arlorio, M., Headspace solid-phase micro extraction coupled

to comprehensive two-dimensional with time-of-flight mass spectrometry applied to the evaluation of Nebbiolo-based wine volatile aroma during ageing. *Int. J. Food Sci. Technol.* **2014,** *49*, 787-796.
68. Du, X.; Song, M.; Baldwin, E.; Rouseff, R., Identification of sulphur volatiles and GC-olfactometry aroma profiling in two fresh tomato cultivars. *Food Chem.* **2015,** *171*, 306-314.
